# Evidence of a Large Novel Gene Pool Associated with Prokaryotic Genomic Islands

**DOI:** 10.1371/journal.pgen.0010062

**Published:** 2005-11-18

**Authors:** William W. L Hsiao, Korine Ung, Dana Aeschliman, Jenny Bryan, B. Brett Finlay, Fiona S. L Brinkman

**Affiliations:** 1 Department of Molecular Biology and Biochemistry, Simon Fraser University, Burnaby, British Columbia, Canada; 2 Department of Statistics, University of British Columbia, Vancouver, British Columbia, Canada; 3 Michael Smith Laboratory, University of British Columbia, Vancouver, British Columbia, Canada; The Institute for Genomic Research, United States of America

## Abstract

Microbial genes that are “novel” (no detectable homologs in other species) have become of increasing interest as environmental sampling suggests that there are many more such novel genes in yet-to-be-cultured microorganisms. By analyzing known microbial genomic islands and prophages, we developed criteria for systematic identification of putative genomic islands (clusters of genes of probable horizontal origin in a prokaryotic genome) in 63 prokaryotic genomes, and then characterized the distribution of novel genes and other features. All but a few of the genomes examined contained significantly higher proportions of novel genes in their predicted genomic islands compared with the rest of their genome (Paired *t* test = 4.43E-14 to 1.27E-18, depending on method). Moreover, the reverse observation (i.e., higher proportions of novel genes outside of islands) never reached statistical significance in any organism examined. We show that this higher proportion of novel genes in predicted genomic islands is not due to less accurate gene prediction in genomic island regions, but likely reflects a genuine increase in novel genes in these regions for both bacteria and archaea. This represents the first comprehensive analysis of novel genes in prokaryotic genomic islands and provides clues regarding the origin of novel genes. Our collective results imply that there are different gene pools associated with recently horizontally transmitted genomic regions versus regions that are primarily vertically inherited. Moreover, there are more novel genes within the gene pool associated with genomic islands. Since genomic islands are frequently associated with a particular microbial adaptation, such as antibiotic resistance, pathogen virulence, or metal resistance, this suggests that microbes may have access to a larger “arsenal” of novel genes for adaptation than previously thought.

## Synopsis

More than 250 microbial genomes have been sequenced to date. A significant proportion of the genes in these genomes have no apparent similarity to known genes and their functions are unknown (i.e., they appear to be novel). As the number of sequenced genomes increases, the number of these novel genes continues to increase. In this paper, the authors now show, through an analysis of a diverse range of prokaryotic genomes, that novel genes are more prevalent in regions called genomic islands. Genomic islands are clusters of genes in genomes that show evidence of horizontal origins. This study is notable since genomic islands disproportionately contain many genes of medical, agricultural, and environmental importance (e.g., animal and plant pathogen virulence factors, antibiotic resistance genes, phenolic degradation genes, etc.). The observation that high proportions of novel genes are also localized to genomic islands suggests that microbes may have access to a larger “arsenal” of novel genes for important adaptations than previously thought. These results also imply that there are different gene pools associated with recently horizontally transmitted genomic regions versus regions that are primarily vertically inherited. The authors suggest that further studies involving large-scale environmental genomic sampling are required to help characterize this understudied gene pool.

## Introduction

Since the publication of the first bacterial genome [1], a consistent observation made by biologists is that a significant portion of a prokaryotic genome encodes putative proteins with no known functions. Even in well-studied free-living microbes such as *Escherichia coli* and *Bacillus subtilis,* more than 35% of their predicted proteomes do not have functional assignment [2,3]. Peer Bork and others have observed that the functions of less than 70% of proteins in unicellular genomes can be predicted with reasonable confidence, a phenomenon which he termed “the 70% hurdle” [4]. Despite the ever increasing number of genomes becoming available, this observation still holds true in the majority of the genomes sequenced. Moreover, the total number of hypothetical genes is steadily increasing as more genomes are sequenced [5]. This suggests that there may be a genetic pool that is being neglected in functional studies of genes to date. With the exploration of sequence data from environmental samples [6,7], scientists have begun to further appreciate the vast number of novel genes in the environment (in particular those with no detectable homologs versus “conserved hypothetical” genes) that appear to be harbored by yet unculturable and unstudied organisms.

In our studies of selected genomic islands (GIs), defined as horizontally acquired genomic regions that may have mutated to obfuscate or destroy their modes of transmission and integration, we anecdotally observed that the distribution of genes annotated as hypothetical in prokaryotic genomes is non-random. The name genomic island is derived from the term pathogenicity island (PAI), originally coined to describe a cluster of virulence genes identified in uropathogenic *E. coli* [8] but not found in closely related strains or species. PAIs have been noted for their important roles in bacterial pathogenesis. For example, the pathogenicity island SPI-2 of *Salmonella typhimurium* encodes a type III secretion system required for intracellular proliferation and systemic infection in a mouse model [9,10]. Mutants of the SPI-2-encoded genes result in attenuation of virulence suggesting that these genes are intricately involved in the infection process [11,12]. Subsequently, genetic elements, which share the same structural features of PAIs, were found in non-pathogenic microorganisms to serve other adaptive functions; these PAI-like elements are collectively referred to as GIs [13]. In the few short years since their discovery, GIs have already been associated with many important adaptive functions that contribute to different microbes' unique life styles. For instance, nitrogen fixation in *Rhizobiaceae* species is encoded by “symbiosis islands”[14], genes for phenolic compound degradation in *Pseudomonas putida* are found on “metabolic islands”[15], and the iron-uptake ability of many pathogens are conveyed by “adaptive islands” [16]. Since GIs have been noted to contribute to a microorganism's fitness, metabolic versatility, and adaptability, we decided to develop a method to computationally identify microbial GIs in a large dataset of completely sequenced microbial genomes and to investigate further what features are noted in these agents of microbial innovation. GIs have been previously detected by the genetic features reported to be associated with them [17] and by comparative genomic and phylogenetic approaches [18,19]. Features reported to be associated with GIs include the presence of flanking repeats, mobility genes (e.g., integrases and transposases), proximal transfer RNAs (tRNAs), and atypical guanine and cytosine content [20]. More recently, we and others have used additional species-specific DNA signatures such as oligonucleotide biases and codon adaptation index to identify GIs [21–25]. However, there is a need to better quantify exactly which features and methods best identify GIs. Then we can use more objective criteria to investigate additional features and properties of these important genomic regions.

In this study, we performed a comprehensive analysis of a dataset of 95 known GIs and related prophages to determine which features best identify these genomic regions. We then used sets of objective criteria based on these features to predict putative GIs on a genome-wide scale for 63 prokaryotic organisms. Through analysis of additional features associated with islands, we found that novel hypothetical genes (genes with no detectable homologs using two independent sequence similarity search methodologies, as discussed below) are significantly more prevalent in GIs versus the rest of the genome, irrespective of what method of novel gene identification is used. From this and additional analyses, we propose that there is a large, separate gene pool associated with such horizontally transferred genomic regions; and this gene pool is a more notable source of innovation in a wide range of taxa involving both bacteria and archaea.

## Results/Discussion

### Prevalence of Features Associated with Reported GIs: Dinucleotide Bias and Associated Mobility Genes Are the Best Predictors

As part of our analysis of features associated with GIs, we created a curated dataset of 95 previously reported horizontally acquired genetic elements (containing 4,553 genes) that we collectively refer to as “known islands” (see Materials and Methods, Table S1). The number of genes in each of these islands ranges from four to 579, reflecting the diversity of these elements. We then inspected each of these known islands for the presence of four GI-associated features (Table 1). This analysis, plus additional analyses of the degree to which each feature overlaps an island (Table S2), indicates that dinucleotide bias is much more sensitive versus conventional %G+C analysis in identifying putative GIs. Using our dataset, the dinucleotide bias approach detected almost three times more of the known islands than the %G+C approach (59 of the islands contain dinucleotide bias versus 23 with %G+C bias). In fact, less than a quarter of the islands examined have abnormal %G+C according to a previously developed cutoff suggesting that by using %G+C alone, many potential GIs may be missed. Only two of the 95 islands examined have abnormally high %G+C, while low %G+C islands are ten times more common. This is in agreement with the observation by Daubin et al. [26,27] that A+T rich genes are preferentially acquired.

**Table 1:**
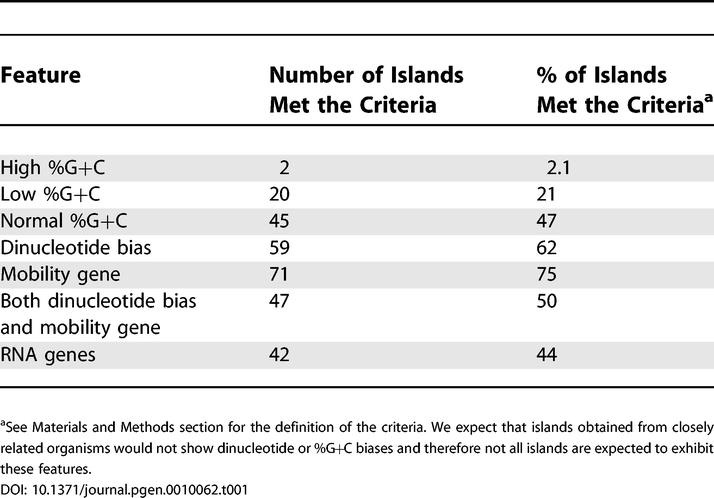
List of GI-Associated Features and the Number and Percentage of Islands Meeting Each Criterion

Highly expressed genes such as ribosomal proteins have been found to exhibit anomalous sequence compositions [28] and can be a source of false positives in dinucleotide bias analysis. We therefore examined the possibility of incorporating other features into our methodology for island detection. Mobility genes and structural RNA (tRNA and tmRNA) genes also appear to be good indicators of horizontal gene transfer (HGT). Of the 41 GIs inspected, 19 have tRNAs suggesting that phage or phage-like elements may be the precursor for these GIs since some phages are noted for using tRNAs as preferred sites for integration [29]. Three-quarters of the islands inspected contain one or more mobility genes making it the most prevalent feature in our dataset. By combining the two best predictors (mobility genes and dinucleotide bias), approximately 50% of the islands satisfy both criteria. Moreover, among approximately 300 predicted islands in our ORF_ALL dataset (see Materials and Methods) we detected only four potential false positives (i.e., ribosomal protein operons with associated mobility genes that may not be HGT).

In subsequent systematic analyses, we predicted islands using criteria that utilized both a dinucleotide bias-based approach alone (the DINUC dataset with higher sensitivity, also referred to as recall in computer science) and a combined dinucleotide bias method and mobility gene identification (the DIMOB dataset with higher specificity, also referred to as precision). By examining our data using both methods, we were able to assess whether trends we examined held true regardless of whether the method favors sensitivity or specificity.

### Overview of Analysis of Predicted GIs: Prevalence of Islands in a Given Microorganism Reflects the Life Styles of the Organism

Our island prediction results are listed in Table S3 (DINUC dataset) and Table S4 (DIMOB dataset). The number of islands and the number of genes in the islands for each of the organisms examined are summarized in Table S5. According to the more precise DIMOB criteria (see Table 2 for summary), 12 organisms did not contain GIs. Most of these organisms are strict intracellular organisms that have restricted access to external gene pools and little or no proposed HGT [30–34], or these organisms appear to be undergoing genome reduction [35]. This supports past statements that HGT occurs more commonly in bacteria that have access to a horizontal gene pool [36].

**Table 2:**
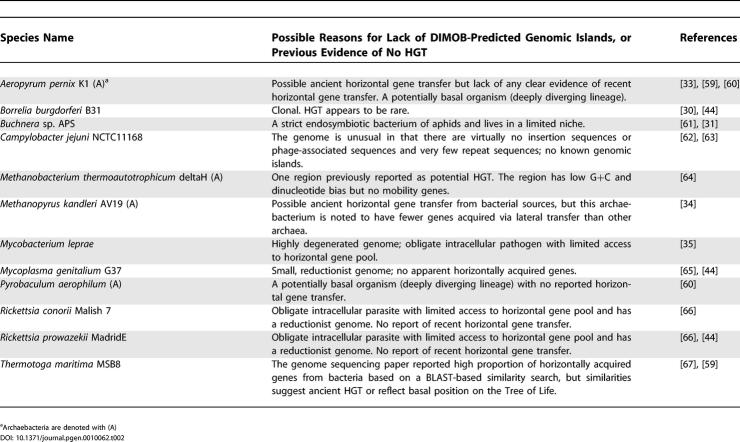
Summary of Organisms without Genomic Islands Based on the DIMOB Criterion

There are some notable limitations to this analysis. Our examination of known islands demonstrates that the DIMOB criterion, though more accurate, does under-identify islands. Furthermore, if a horizontally acquired region shares similar sequence composition features with the host sequence, no composition-based approaches will detect such GIs. This may explain why there were few islands detected in *Neisseria meningitidis* despite their natural competency for DNA uptake and exchange and their lack of clonality. *Neisseriaceae* are noted for horizontal DNA exchange [37]; however, this exchange occurs primarily between *Neisseria* species, which have similar genome sequence compositions. Our analysis represents an examination of HGT between more distantly related organisms (or gene sources) that have different sequence compositions.

### Comparative Analysis of GIs versus Non-GIs: Distributions of Gene Function Categories Differ

It has been proposed that certain types of genes are more likely to be horizontally transferred. For example, based on a phylogenetic analysis, Jain et al. observed that informational genes (translation and transcription) are far less likely to be horizontally transferred than operational (housekeeping) genes [38]. A recent paper by Nakamura et al. [24] also found cell surface, DNA binding, and pathogenicity-related genes to be more prevalent in horizontally acquired regions. We analyzed all organisms in our dataset to see if the distributions of protein function categories differ for proteins encoded by genes in islands versus those outside of islands. Genes were classified into 22 clusters of orthologous groups of proteins (COG) functional categories plus a “none” category for proteins without COG assignments (i.e., the “none” category implies that the protein does not have three or more orthologs in other species and so is a relatively “novel” gene).

**Figure 1:**
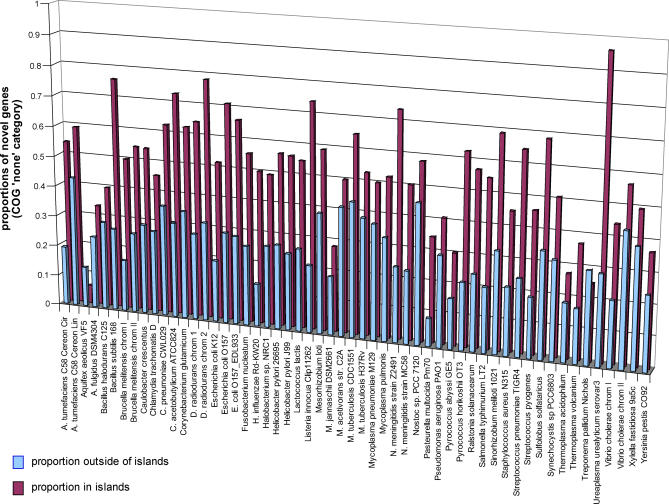
Proportion of Novel Genes in Genomic Islands (Red Bars) versus the Rest of the Genome (Blue Bars) according to a COG-Based Analysis Proportions of novel genes are calculated as a percentage of all genes within islands or outside of islands, respectively, for each genome (listed on the x axis). A paired *t* test indicates that significantly more genes in islands versus non-islands do not have a COG classification (*p* = 1.20E-18). This phenomenon is uniform across prokaryotic lineages and domains. Similar results are also observed if different datasets are analyzed, or different methods for identifying novel genes are used (Table 3).

The most striking observation was that the proportion of genes in the “none” category, which, for readability, we refer to as “proportion of novel genes,” was higher in islands than outside for almost all organisms. On average, 42% of the genes in islands are novel compared to 26% of the genes outside of islands for an organism using the DINUC criteria. The result for the DIMOB dataset is also consistent (53% for islands genes and 28% for outside genes). The actual proportions do vary widely between organisms (though the general trend is consistent) so caution is required when interpreting the means. Figure 1 shows the pair-wise comparison for each organism in the DIMOB dataset and Table S6 tabulates the results for all criteria examined. This observation of a higher proportion of novel genes in islands was statistically significant regardless of whether the DINUC (Paired *t* test, *p*-value = 1.27E-18) or the DIMOB (*p*-value = 1.20E-18; Figure 1) criterion was used to define putative GIs. Since this observation has not been rigorously validated in the past, we decided to characterize and validate this observation further. The other category of genes that is over-represented within islands in both sets of predicted islands (DINUC and DIMOB) is the genes involved in DNA replication, recombination, and repair. Conversely, genes involved in macromolecule biosynthesis (transport and metabolism genes for lipid, amino acid, nucleotide, carbohydrate, and co-enzymes) are present in significantly lower proportions in the predicted islands versus outside of islands. We also found that there is no difference in the proportion of transcriptional genes in islands versus outside of islands. This result may appear to contradict observations made by Jain et al. [38]; however, the differences are likely due more to differences in the type of HGT being detected. Their dataset from six organisms consisted of a rather small set of homologous genes which were more likely to be subject to orthologous displacement than to de novo acquisition. Since sequence compositional approaches are less able to detect orthologous displacement from organisms with similar compositions, our results suggest that if these genes have indeed undergone HGT, the mode of transfer is likely to be homologous recombination between closely related species or ancient HGT that has been subject to amelioration. See Protocol S1 for details regarding this analysis and Table S7 for the tabulated results.

**Table 3:**
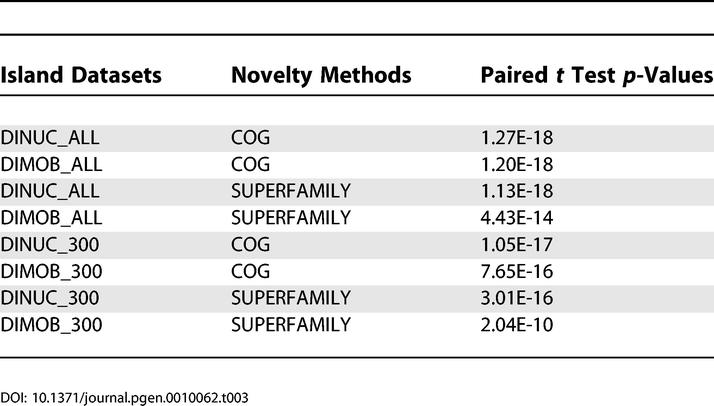
Summary of *p-*Values Using Different Datasets and Methods

Notably, unlike the “none” category, there is no difference in the proportions of genes in the “general function prediction” and “unknown function” COG categories. These two categories primarily consist of genes encoding conserved hypothetical proteins (i.e., proteins of unknown function that are not “novel” to a given species). This implies that there is not necessarily a bias in terms of what conserved genes have been functionally studied in GIs versus non-GIs to date. The increase in hypothetical genes in GIs is primarily due to the increased occurrence of novel, relatively unconserved genes in these genomic regions.

### Higher Proportions of Novel Genes in Predicted GIs Are Independent of the Method Used to Identify Novel Genes

**Figure 2:**
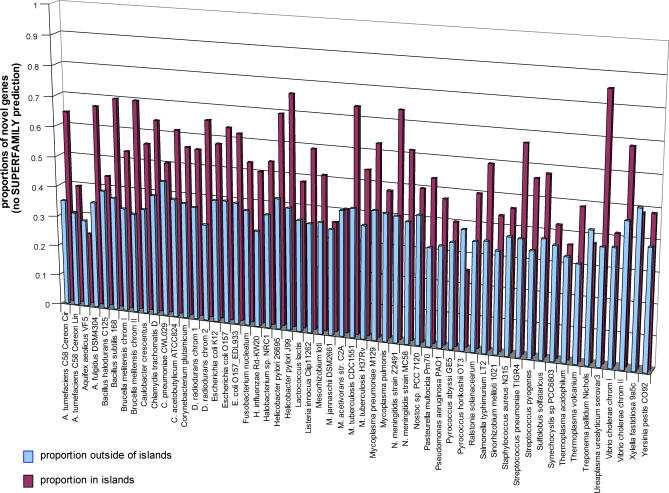
Proportion of Novel Genes in Genomic Islands (Red Bars) versus the Rest of the Genome (Blue Bars) according to a SUPERFAMILY-Based Analysis Proportions of novel genes are calculated as a percentage of all genes within islands or outside of islands, respectively, for each genome (listed on the x axis). A paired *t* test indicates that significantly higher proportions of genes in islands (red bars) versus outside islands (non-islands; purple bars) do not have a SUPERFAMILY prediction (potential novel genes; *p* = 4.43E-14).

COG analysis is suitable for detecting orthologous genes but may fail to identify more “distant” homologs that have complex evolution histories (e.g., multiple duplications and deletions among lineages). To complement the COG-based analysis of novel genes in GIs, we adapted another independent method to detect novel hypothetical proteins called SUPERFAMILY analysis [39]. We chose this method because of its reported accuracy and its ability to detect more remote homologs based on structurally conserved similarities [40]. Genes that cannot be assigned to a SUPERFAMILY do not have detectable structural domain homologs in the SCOP database, and therefore are more likely to be novel genes. In both the DINUC and DIMOB datasets, we observed that the proportion of such novel genes is significantly higher in islands compared with outside of islands (see Table 3 for a list of *p*-values from paired *t* tests). Pair-wise comparison for the DIMOB dataset is illustrated in Figure 2 and tabulated in Table S6 together with the other datasets. This SUPERFAMILY analysis, like the COG-based analysis, indicates that the vast majority of microbes examined have more novel genes in GIs. The SUPERFAMILY analysis, however, may be considered to be more rigorous and subject to less sampling bias in the Tree of Life than the COG-based analysis, because it can detect more distantly related homologs. Notably, the proportion of novel genes outside of islands according to the SUPERFAMILY analysis is remarkably consistent regardless of the lineages of the organisms. Any variability in novel gene content between organisms does appear to primarily occur in GI regions.

### Higher Proportions of Novel Genes in Predicted GIs Are Not Due to Less Accurate Gene Prediction in GI Regions

One possible explanation for the higher proportion of novel genes in GIs is that genes in GI regions are more frequently mispredicted. Most commonly used gene prediction algorithms today incorporate genomic composition measures such as codon usage to aid in the identification of genes. They also require training with a subset of known genes in an organism in order to become familiar with that organism's genomic composition [41,42]. Gene prediction could presumably be failing more frequently in GI regions because of their differing genomic compositions that lead to more false predictions of genes and consequently more predicted “novel” genes in these regions. Also, since our method of calculating dinucleotide bias uses gene clusters rather than sliding windows of a fixed size (in base pairs), shorter genes may reduce the sampling size to the point of increasing the chance of biased sampling. We addressed this issue by re-examining our data after removal of open reading frames (ORFs) less than 300 bps from our gene sets for each organism. Since longer ORFs are more likely to encode truly functional genes (see Materials and Methods), this reduces the probability that a novel gene is falsely predicted. We used the same two criteria for GI identification, namely dinucleotide bias alone and dinucleotide bias plus mobility gene identification, to generate two lists of islands, which we called the “DINUC_300” and “DIMOB_300” datasets respectively. Because the island detection process was carried out after genes less than 300 bps were removed, this resulted in slightly different lists of the number of islands (see Table S5), as well as which genes were present in each island. Despite these adjustments, the proportion of novel genes in islands was still statistically significantly higher (compared to outside of islands) for both the COG- and SUPERFAMILY-based analyses using either the DINUC or DIMOB dataset (results tabulated in Table S8). Pair-wise *t* tests for these analyses ranged from a *p*-value of 2.04E-10 to 1.05E-17 (for complete list, see Table 3). These *p*-values, while still highly significant, are slightly higher than the ones derived from full gene set. This variation suggests that the ORFs less than 300 bps in length do influence the analysis, but that the contribution is very minor since the *p*-value is still very significant. We therefore conclude that the over-representation of novel genes in GIs is not predominantly due to more falsely predicted genes in such regions. There appears to be a genuine increase in the number of novel genes in GIs.

### Higher Proportions of Novel Genes in Islands Are Not Due to Domain Coverage Bias of COG and SUPERFAMILY

COGs are constructed from fully sequenced microbial genomes which contain prophages and phage-like elements, but exclude plasmids, phages, and other extrachromosomal elements. Therefore COG may have better coverage of the domain comprising prokaryotic chromosomal proteins versus phage and plasmid-associated proteins. SUPERFAMILY, which is based on the SCOP structural classification database, and therefore includes proteins from all domains of life, albeit at different ratios, may be less subject to this bias. Since phage and plasmid mobile elements are potential sources of HGT, higher proportions of novel genes in our DINUC and DIMOB islands may be due to the coverage bias of the methodologies. To investigate this, we searched all of the translated products of the novel genes in and outside of islands against proteins encoded by prokaryotic plasmids and phage genomes using BLAST. With the criteria and database we used, we would expect some novel genes to encode homologs of plasmid and phage-associated proteins, but we wished to discover whether they would be disproportionately associated with genomic islands or not. The results showed that while some of the novel genes did indeed have detectable homologs in our plasmid and phage dataset (Table 4), the majority do not. Moreover, the proportions of novel coding genes with similarity to plasmid and phage proteins are almost identical in the DINUC islands and outside of islands (~30%, see Table 4). For the DIMOB islands, since the dataset is enriched with elements that are more likely to have phage and plasmid origins (by incorporating transposases and integrases as part of the definition of islands and by reducing the number of potential false positives, such as highly expressed genes not associated with these mobile elements), we would expect to see an enrichment of genes with phage and plasmid homologs. Indeed, this is what we observed (Table 4). However, even after taking this potential bias into account by down-adjusting the novel gene counts in DIMOB islands by 11.5% (40.47% minus 28.98%), the proportion of novel genes in islands is still significantly higher than outside (the paired *t* test *p* value is 4.76E-16). Therefore, we can conclude that while COG and SUPERFAMILY searches missed some phage or plasmid encoded genes, this omission does not significantly contribute to the observation of higher proportion of novel genes in islands. Notably, the observation suggests that the current sampling of plasmids and phage genomes, which are mostly from culturable prokaryotic hosts, does not account for most of the horizontal gene pool contributing to islands.

**Table 4:**
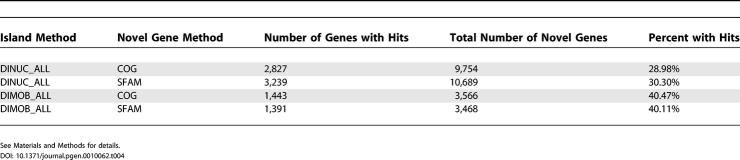
Proportions of Novel Genes with BLAST Hits in the Phage and Plasmid Database at Expect Value Cutoff of 1E-5

### Higher Proportion of Novel Genes in Islands Is Statistically Significant in Many Organisms while the Reverse Is Never Observed

We further assessed the proportion of novel genes at the level of an individual organism (for organisms with more than one chromosome, though each chromosome was analyzed independently). We used a chi-square test of independence or Fisher's exact test (when the number of novel genes in islands is small) to see whether the proportion of novel genes is the same for the within-GI gene pool and for the outside-GI gene pool. Our results (summarized in Table 5) showed that regardless of the GI prediction criteria (DINUC or DIMOB) or the novel gene prediction method (COG or SUPERFAMILY) used the majority of organisms show significantly higher proportion of novel genes in islands. Even after taking multiple testing into account by drastically adjusting the *p*-values upward using the Bonferroni correction, the observation still holds true. While this biased occurrence of novel genes in GIs is relatively independent of the prokaryotic lineage examined, certain organisms do not exhibit statistically higher proportions of novel genes in islands. This may reflect a genuine reduced access to our described novel gene pool (e.g., hyperthermophilic microorganisms at the base of the Tree of Life may have reduced access to the relevant phage, which as we discuss below, is a probable source of HGT), or it may simply reflect a bias in analysis of organisms with few close relatives. In the latter case, however, one would expect that the proportion of novel genes in non-island regions to be higher, reflecting a lack of similarity to genes in other organisms. However, visual examination of both Figures 1 and 2 indicates that the proportion of novel genes in non-island regions is not notably higher for those organisms with insignificant novel gene bias. It is intriguing to note that organisms with an observed lower proportion of novel genes in islands never achieved statistical significance. This further confirms that the source of genetic material for the GIs analyzed is different and less well characterized than the source of the more stable “core” genome.

**Table 5:**
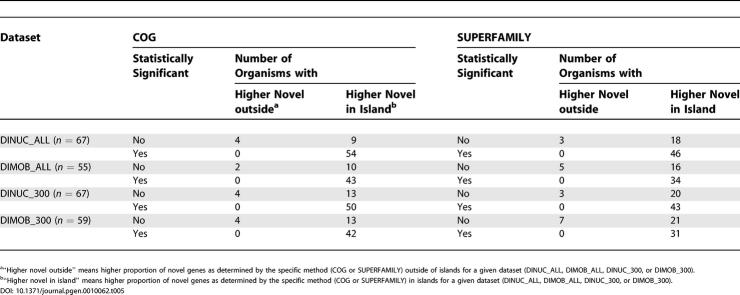
Number of Organisms Distributed by Proportion of Novel Genes and Statistical Test Significance

### Further Analysis of the Proteins Encoded in Islands Indicates that Their Subcellular Localization Distribution Is Different, and Similar to Phage

We used PSORTb version 2.0 [43] for de novo prediction of bacterial protein subcellular localization in order to gain insight into whether genes in islands encode proteins with preferential localizations. PSORTb currently generates the most precise predictions available (more than 95% precision) with similar recall as other methods. Biases toward particular predicted subcellular localizations could provide further clues regarding the origin and function of these novel predicted proteins. We found (Table 6) that proteins encoded in islands are less often predicted to be localized to the cytoplasmic membrane (CM) of both Gram-negative and Gram-positive bacteria (Paired *t* tests ~5.0E-7 and 8.7E-9, respectively). This is notable, because CM proteins can be predicted with very high accuracy (more than 95% precision and recall). There have been indications that some GIs are of phage origin [26], therefore we performed an additional analysis of CM proteins associated with phage by examining the subcellular localization of deduced proteins from annotated phage or phage-associated genes in *E. coli* and *B. subtilus* genomes. We found that the proportion of integrated phage proteins predicted to be in the CM is lower versus other proteins for such organisms. Only 4–5% of phage-associated proteins are predicted to target the CM compared to 15–20% of bacterial genes. These observations further support proposals that phages may be the source of HGT in prokaryotic organisms.

**Table 6:**
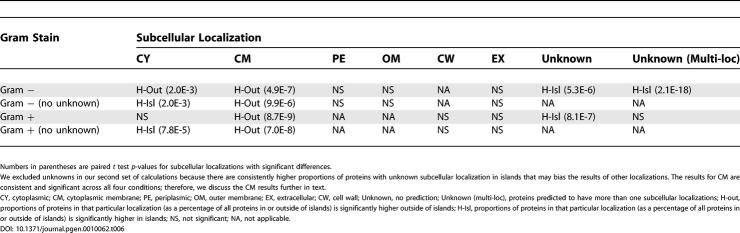
Distribution of Predicted Subcellular Localization of Proteins in Islands Compared to Outside of Islands

### Implication of Higher Proportions of Novel Genes in Islands: The Big Picture

HGT has been found to be a formidable force in prokaryotic innovation [44]. In this study, we first evaluated several GI-associated features and determined that dinucleotide bias and mobility genes are more sensitive indictors of HGT than the more commonly used %G+C anomaly. Using these indicators, we then constructed a couple of objective criteria to define putative GIs. We have now shown through the largest, most comprehensive analysis of its kind, that novel genes are more likely to be present in GIs and related horizontally acquired regions, than in the rest of a prokaryotic genome. This biased distribution is observed on the majority of taxa examined to date and does not appear to be an artifact of false gene prediction. Moreover, we have shown that the reverse (i.e., higher proportions of novel genes outside of islands) does not reach statistical significance. Also, conserved hypothetical genes do not have a similar distribution bias, suggesting that this observation is not more generally associated with genes of unknown function, but rather is specific to genes that are relatively novel, or specific, to a lineage. Clearly our analysis only detects and examines a subset of horizontally acquired genes as horizontal acquisition from organisms with similar genome sequence compositions and ancient HGT would not be detected by our methods. However, significant implications can still be drawn from our observations.

First, the gene content associated with GIs and related regions is significantly different from gene content in other regions. This implies that the gene pool associated with such GI elements have different composition and characteristics. Furthermore, the increased prevalence of novel genes in such regions suggests that the associated gene pool may be larger, or otherwise subject to more innovation, than in the vertical gene pool. In a recent study by Lerat et al. [45], they took a phylogenetic approach to look at genomic repertoires of gamma-proteobacteria and noticed that single unique genes within the phyla are predominately most parsimoniously explained by HGT from distant sources rather than gene duplication and loss. Based on this and other observations, Lerat et al. also suggested a large pool of available genes for gamma-proteobacteria. Our study indicates that this observation is universal to a wide range of prokaryotes—both from the *Bacteria* and *Archaea* domains of life. In this context it is therefore perhaps ominous that GIs are noted for their association with particular adaptations of a microbe, such as antibiotic resistance or pathogen virulence. Significantly higher proportion of novel genes in GIs therefore cautions us that microbes may have a larger “arsenal” of novel genes for adaptation to environments, including resistance to anti-microbial approaches, than previously thought.

We also noted that other biased features of genes in GIs, such as the subcellular localization of their deduced proteins, were consistent with phage. There is increasing evidence that phage and GIs are related [26,46]. While transformation, transduction, and conjugation all have been implicated as mechanisms for HGT, recent analyses have indicated that phage transduction is the predominant force in cross-taxa transfer [29]. With phage diversity approximately ten times that of the prokaryotic diversity, several researchers have proposed that phage can contribute to the genetic individuality of bacterial strains at a much higher level than previously believed [47]. Our results support this, and further support that there is a gene pool with considerable diversity, potentially related to phage, that affects a wide diversity of bacteria of medical and economic importance, as well as archaea. Furthermore, our results pointed out that sampling phages, commonly associated with culturable prokaryotes, are insufficient to elucidate this diverse gene pool and that additional sources still need to be characterized. Metagenomic approaches of environmental samples may provide further clues regarding the nature of these sources.

This work is consistent with the hypothesis that genomes are composed of a more stable set of “core” genes and adaptive “life style” genes [48]. Since genomic sequences only provide snapshots of an organism's evolutionary history, the fate of these “life style” genes is largely unknown. Some evidence suggests that at least some of these life style genes are maintained and have become useful to the hosts [27,45]. Having the ability to draw novel genes from the environment to satisfy short-term needs provides an economical and effective strategy for survival. By characterizing these genes, we can gain new insights into what makes an organism unique and able to adapt to its current environment. While headway has been made in the characterization of conserved hypothetical proteins [49], our most valuable in silico tools for protein characterization are still predominantly based on sequence similarity. Little success has been achieved in de novo characterization of hypothetical proteins. As a result, studying novel hypothetical genes in silico or at the bench provides a significant challenge to researchers. Our inability to effectively functionally characterize these novel hypothetical genes may hamper our ability to devise tailored strategies to combat different microbial pathogens and resistance mechanisms.

Recent efforts focused on environmental genomic sampling and metagenomic projects [6,50,51] will help us obtain sequences that may elucidate the sources of these novel hypothetical genes from organisms whose genomes have yet to be sequenced. Our results provide a strong rationale for the continuation of these efforts. Regardless of the source of this innovation, it appears that novel genes are being acquired by prokaryotes disproportionately through GIs. A wide range of microbial research areas are impacted by this adaptation strategy due to the association between GIs and microbial adaptations of importance.

## Materials and Methods

### Genome sequence data and organisms examined.

Sequence and annotation of each ORF of completely sequenced prokaryotic genomes were downloaded from the National Center for Biotechnology Information (NCBI) FTP site in April, 2004. We limited our final dataset to the 63 organisms used in the most recent publication that analyzed COG [52] because we wished to adopt the most consistent and accurate COG dataset in our analysis. The selection of 63 organisms, nevertheless, represents a wide range of taxa [52]. We examined only chromosomal sequences, not plasmid data. To reduce the number of falsely predicted ORFs and to avoid sequence compositional bias due to short ORFs, we also constructed a separate dataset by excluding any ORFs smaller than 300 bps. We labeled the first set of ORFs containing all the predicted ORFs, “ORF_ALL,” and the second set “ORF_300.”

### GI dataset development and validation of GI features.

We constructed a verified dataset of 41 known GIs and 54 prophages from 14 well-studied organisms (ten species) through a manual literature research (Tables S1 and S2). We then examined the prevalence of four sequence and annotation features commonly reported with GIs. These four features (%G+C bias, dinucleotide bias [53], presence of tRNA genes, and presence of mobility genes) were identified using our previously developed IslandPath software [21]. For each available prokaryotic genome, IslandPath generates a graphic representation of the genome and superimposes these features on the image. The genetic elements in our dataset were inspected manually, using the IslandPath analysis, for the presence or absence of the four GI-associated features. For mobility and tRNA genes, an island is scored positively if it contains at least one gene annotated as such. An island is considered to exhibit %G+C or dinucleotide bias if more than half of the ORFs in that island have these biases as determined by IslandPath [21]. ORFs with %G+C more than 4.62% above or below the genome average are marked as “High %G+C” or “Low %G+C”, respectively. All the other ORFs are noted as “Normal %G+C.” We derived this cutoff from a previous study of genome %G+C variation in obligate intracellular bacteria that are thought to be subject to little horizontal gene transfer [36]. This cutoff is thought to reflect the inherent %G+C variation of an organism due to other factors such as gene expression level [36].

### GI prediction.

Based on our GI feature validation results, we defined a putative GI as eight or more consecutive ORFs with dinucleotide bias (DINUC dataset), or eight or more consecutive ORFs with dinucleotide bias plus at least one mobility gene present in the region (DIMOB dataset). Mobility genes were identified using the NCBI annotation and PFAM hidden Markov models (HMMs) searches. The PFAM HMM search was conducted as follows: in order to identify putative mobility genes in a large number of genomes in a reasonable amount of time, we used the Paracel GeneMatcher system, a hardware-based solution for carrying out similarity search in parallel. To further speed up the search, instead of searching all of the predicted ORFs against all of the PFAM HMMs, we identified and searched against 46 PFAM HMMs representing mobility genes (e.g., integrases and transposases). Results with expect values (similar to BLAST E-value) smaller than 0.01 were retained. Manual inspection of results from a randomly selected set of five species did not reveal any obvious false positives using this cutoff. Genomic regions that satisfied the above criteria were extracted and the genes in these regions were labeled as “islands.” The rest of the genes were labeled as “outside of islands.”

We also performed a prediction of GIs after removal of all genes that are less than 300 bps in length (ORF_300) to reduce the possible impact of incorrectly predicted small genes on our analysis. We chose a 300-bps cutoff (corresponding to 100 amino acids) because we and others have previously found, through comparisons of the genome-wide gene predictions of closely related organisms not subject to much HGT, that annotation of genes shorter than this cutoff by separate groups becomes more inconsistent [36]. In addition, a 300-bps cutoff has been commonly used for some genome annotation processes [33].

### Functional characterization of genes in GIs.

To avoid inconsistencies in genome annotation from different sequencing projects, we used two independent bioinformatic tools to assign ORFs to different functional categories. We chose COG and SUPERFAMILY [54] because of their complementarities. COG is suitable for predicting “closely related” homologs which are likely to be orthologous because a COG is defined as three or more proteins that all share the highest sequence similarity with each other. Detailed description of how a COG was constructed and subsequently updated can be found in [55] and [52] SUPERFAMILY, on the other hand, provides functional assignments to protein sequences at the superfamily level of the SCOP protein structural classification system [56]. Proteins in a SCOP superfamily are likely to share a common evolutionary origin based on their structural similarities. As a result, the SUPERFAMILY predictors are useful in detecting more remote homologs that have similar structural features. Both programs have been shown to make reliable assignments and have been widely used [40,57,58]. COG assignment results were obtained from the NCBI FTP site. We used the dataset published with the updated COG paper rather than the subsequent assignments associated with the NCBI genome “ptt” files since there appears to be some inconsistency and omissions of COG assignments in these files. Pre-computed SUPERFAMILY genome assignment results (Version ass_09 May, 2004) were obtained, with permission, from Julian Gould, the original lead author of the SUPERFAMILY database. With both COG and SUPERFAMILY assignments, we used the cutoffs set by the respective authors for filtering out non-significant hits. For SUPERFAMILY, the expect value cutoff used was 0.02 (provided by the authors of the database). Since we are not trying to identify specific functions using SUPERFAMILY and can tolerate some false assignments, this more relaxed cutoff seems adequate. For COG, the cutoff(s) used is not reported and as argued by the authors of the COG database, the absolute cutoff is not crucial since all COGs have to satisfy the “best BLAST hit to multiple other organisms” constraint.

The phage genome and plasmid records were obtained from the NCBI Entrez Genome site (http://www.ncbi.nih.gov/entrez/query.fcgi?db=Genome) in September, 2005. There are 284 phage genomes and 716 plasmids records. Protein FASTA records associated with these genomic sequences were downloaded by following the NCBI Protein Linkouts of these records. These protein records were converted into a local BLAST database and searched against using the NCBI BLASTP program. Queries (translated products of either COG-based or SUPERFAMILY-based novel genes) that have database matches with an expect value less than 1E-5 were considered to have homologs in this phage and plasmid database.

PSORTb version 2.0 [43] was used to predict protein subcellular localization for deduced proteins from all complete genomes analyzed. Custom Perl scripts were used to combine records obtained from various sources and to link annotations.

### Statistical analyses.

Each COG functional category was assessed for over- and under-representation in predicted GIs across all species. This was done by first expressing the number of genes in a category as a percentage of all the genes in islands for a given organism. The percentage of genes outside of islands for the same category was likewise calculated. We calculated the two percentages for each organism and for each category including a “none” category into which we assigned genes without a COG category. For each category, we could then determine if the genes in that category are over- or under-represented in islands through a paired *t* test analysis (in island versus outside island) across all organisms. We carried out the same *t* test analysis to determine whether genes lacking a SUPERFAMILY prediction are over-represented in island across all organisms. For each organism, we also determined if the proportions of “novel hypothetical” genes (genes without COG or SUPERFAMILY assignments) in islands are significantly different from those outside of islands using chi-square test of independence. In a few cases where the numbers of these novel hypothetical genes are small in islands, we used Fisher Exact test instead. We considered *p*-values smaller than 0.05 to be significant. Statistical analyses were done using *R* statistics package.

## Supporting Information

Protocol S1 Additional Analysis and Discussion Regarding COG Categories (22 KB DOC)

Table S1 List of Reported and Known Genomic Islands and Phages (28 KB XLS)

Table S2 List of Reported and Known Genomic Islands and Phages (Graded) (24 KB XLS)

Table S3 List of Genes in Predicted Genomic Islands (Criterion: DINUC_ALL) (3187 KB XLS)

Table S4 List of Genes in Predicted Genomic Islands (Criterion: DIMOB_ALL) (903 KB XLS)

Table S5 Number of Islands and Number of Genes in Islands by Organisms (25 KB PDF)

Table S6 Summary of Gene Counts in Islands and outside of Islands (All ORFs Included) (27 KB PDF)

Table S7 Gene Count in Each of the COG Categories (Excluding the None Category) (73 KB PDF)

Table S8 Summary of Gene Counts in Islands and outside of Islands (ORFS < 300 bps Excluded) (29 KB PDF)

## Abbreviations

bp: base pair
CM: cytoplasmic membrane
COG: clusters of orthologous groups of proteins
GI: genomic island
HGT: horizontal gene transfer
HMM: hidden Markov models
ORF: open reading frame
PAI: pathogenicity island
