# Supplementary material for: Evidence of a Large Novel Gene Pool Associated with Prokaryotic Genomic Islands
Source: PLoS Genet. 2005 Nov 18;1(5):e62. doi: 10.1371/journal.pgen.0010062 (PMC1285063; doi:10.1371/journal.pgen.0010062)
Supplement: Supplementary file 1 [file pgen.0010062.st005.pdf]

Table S5: Summary of Number of Islands and Number of Genes in Islands by Organisms

DINUC Islands (All ORFs included)

Organism Name	Num of Islands	Num of Genes in Islands
Aeropyrum pernix K1	23	326
Agrobacterium tumefaciens C58 Cereon Circular	21	328
Agrobacterium tumefaciens C58 Cereon Linear	11	165
Aquifex aeolicus VF5	16	193
Archaeoglobus fulgidus DSM4304	29	339
Bacillus halodurans C125	29	429
Bacillus subtilis 168	24	545
Borrelia burgdorferi B31	12	146
Brucella melitensis choromosome I	10	207
Brucella melitensis choromosome II	11	127
Buchnera sp. APS	7	88
Campylobacter jejuni NCTC11168	15	170
Caulobacter crescentus	45	568
Chlamydia trachomatis D	14	145
Chlamydophila pneumoniae CWL029	11	124
Clostridium acetobutylicum ATCC824	47	565
Corynebacterium glutamicum	23	410
Deinococcus radiodurans chrom 1	29	308
Deinococcus radiodurans chrom 2	4	54
Escherichia coli K12	38	549
Escherichia coli O157	56	909
Escherichia coli O157_EDL933	48	866
Fusobacterium nucleatum subsp. Nucleatum ATCC25586	27	287
Haemophilus influenzae Rd-KW20	13	178
Halobacterium sp. NRC1	24	305
Helicobacter pylori 26695	5	110
Helicobacter pylori J99	7	124
Lactococcus lactis	23	310
Listeria innocua Clip11262	31	448
Mesorhizobium loti	66	903
Methanobacterium thermoautotrophicum deltaH	17	217
Methanococcus jannaschii DSM2661	22	269

Methanopyrus kandleri AV19	23	304
Methanosarcina acetivorans str. C2A	60	699
Mycobacterium leprae	14	160
Mycobacterium tuberculosis CDC1551	41	487
Mycobacterium tuberculosis H37Rv	42	499
Mycoplasma genitalium G37	6	62
Mycoplasma pneumoniae M129	9	102
Mycoplasma pulmonis	10	111
Neisseria meningitidis serogroup A strain Z2491	17	242
Neisseria meningitidis serogroup B strain MC58	16	286
Nostoc sp. PCC 7120	67	760
Pasteurella multocida Pm70	16	203
Pseudomonas aeruginosa PAO1	60	690
Pyrobaculum aerophilum	29	348
Pyrococcus abyssi GE5	22	259
Pyrococcus horikoshii OT3	17	202
Ralstonia solanacearum	40	471
Rickettsia conorii Malish 7	18	198
Rickettsia prowazekii MadridE	11	127
Salmonella typhimurium LT2	39	615
Sinorhizobium meliloti 1021	34	443
Staphylococcus aureus subsp. Aureus N315	25	320
Streptococcus pneumoniae TIGR4	12	152
Streptococcus pyogenes	16	206
Sulfolobus solfataricus	38	465
Synechocystis sp PCC6803	27	339
Thermoplasma acidophilum	15	231
Thermoplasma volcanium	18	246
Thermotoga maritima MSB8	19	237
Treponema pallidum Nichols	13	150
Ureaplasma urealyticum serovar3	5	78
Vibrio cholerae chromosome I	21	304
Vibrio cholerae chromosome II	10	201
Xylella fastidiosa 9a5c	26	375
Yersinia pestis CO92	38	459
Total	1632	21743

DINUC Islands (ORFs < 300bps excluded)

Organism Name	Num of Islands	Num of Genes in Islands
Aeropyrum pernix K1	23	310
Agrobacterium tumefaciens C58 Cereon Circular	20	296
Agrobacterium tumefaciens C58 Cereon Linear	11	156
Aquifex aeolicus VF5	19	219
Archaeoglobus fulgidus DSM4304	25	273
Bacillus halodurans C125	26	386
Bacillus subtilis 168	18	433
Borrelia burgdorferi B31	11	140
Brucella melitensis choromosome I	12	186
Brucella melitensis choromosome II	11	131
Buchnera sp. APS	6	74
Campylobacter jejuni NCTC11168	14	162
Caulobacter crescentus	42	505
Chlamydia trachomatis D	13	134
Chlamydophila pneumoniae CWL029	12	131
Clostridium acetobutylicum ATCC824	34	450
Corynebacterium glutamicum	23	366
Deinococcus radiodurans chrom 1	27	303
Deinococcus radiodurans chrom 2	4	49
Escherichia coli K12	29	451
Escherichia coli O157	41	755
Escherichia coli O157_EDL933	39	703
Fusobacterium nucleatum subsp. Nucleatum ATCC25586	28	285
Haemophilus influenzae Rd-KW20	12	161
Halobacterium sp. NRC1	21	264
Helicobacter pylori 26695	7	116
Helicobacter pylori J99	6	104
Lactococcus lactis	24	282
Listeria innocua Clip11262	29	392
Mesorhizobium loti	62	800
Methanobacterium thermoautotrophicum deltaH	16	235
Methanococcus jannaschii DSM2661	21	254
Methanopyrus kandleri AV19	21	271
Methanosarcina acetivorans str. C2A	51	622

Mycobacterium leprae	13	151
Mycobacterium tuberculosis CDC1551	38	449
Mycobacterium tuberculosis H37Rv	35	426
Mycoplasma genitalium G37	7	84
Mycoplasma pneumoniae M129	9	102
Mycoplasma pulmonis	9	102
Neisseria meningitidis serogroup A strain Z2491	14	183
Neisseria meningitidis serogroup B strain MC58	12	199
Nostoc sp. PCC 7120	67	727
Pasteurella multocida Pm70	15	189
Pseudomonas aeruginosa PAO1	55	639
Pyrobaculum aerophilum	25	279
Pyrococcus abyssi GE5	20	240
Pyrococcus horikoshii OT3	16	197
Ralstonia solanacearum	38	452
Rickettsia conorii Malish 7	11	130
Rickettsia prowazekii MadridE	9	106
Salmonella typhimurium LT2	35	527
Sinorhizobium meliloti 1021	27	381
Staphylococcus aureus subsp. Aureus N315	18	243
Streptococcus pneumoniae TIGR4	10	125
Streptococcus pyogenes	15	182
Sulfolobus solfataricus	38	441
Synechocystis sp PCC6803	23	295
Thermoplasma acidophilum	11	216
Thermoplasma volcanium	15	225
Thermotoga maritima MSB8	19	229
Treponema pallidum Nichols	13	140
Ureaplasma urealyticum serovar3	5	68
Vibrio cholerae chromosome I	18	267
Vibrio cholerae chromosome II	8	148
Xylella fastidiosa 9a5c	18	268
Yersinia pestis CO92	34	401
Total	1458	19210

DIMOB Islands (all ORFs included)

Organism Name	Num of Islands	Num of Genes in Islands
Aeropyrum pernix K1	0	0
Agrobacterium tumefaciens C58 Cereon Circular	1	50
Agrobacterium tumefaciens C58 Cereon Linear	3	71
Aquifex aeolicus VF5	1	17
Archaeoglobus fulgidus DSM4304	2	21
Bacillus halodurans C125	8	201
Bacillus subtilis 168	5	267
Borrelia burgdorferi B31	0	0
Brucella melitensis choromosome I	6	112
Brucella melitensis choromosome II	3	46
Buchnera sp. APS	0	0
Campylobacter jejuni NCTC11168	0	0
Caulobacter crescentus	5	61
Chlamydia trachomatis D	1	11
Chlamydophila pneumoniae CWL029	1	8
Clostridium acetobutylicum ATCC824	5	85
Corynebacterium glutamicum	6	154
Deinococcus radiodurans chrom 1	4	56
Deinococcus radiodurans chrom 2	1	23
Escherichia coli K12	15	275
Escherichia coli O157	21	415
Escherichia coli O157_EDL933	24	486
Fusobacterium nucleatum subsp. Nucleatum ATCC25586	3	36
Haemophilus influenzae Rd-KW20	1	12
Halobacterium sp. NRC1	7	138
Helicobacter pylori 26695	2	62
Helicobacter pylori J99	2	50
Lactococcus lactis	11	179
Listeria innocua Clip11262	4	100
Mesorhizobium loti	18	344
Methanobacterium thermoautotrophicum deltaH	0	0
Methanococcus jannaschii DSM2661	2	25
Methanopyrus kandleri AV19	0	0
Methanosarcina acetivorans str. C2A	20	244

Mycobacterium leprae	0	0
Mycobacterium tuberculosis CDC1551	7	91
Mycobacterium tuberculosis H37Rv	9	104
Mycoplasma genitalium G37	0	0
Mycoplasma pneumoniae M129	1	8
Mycoplasma pulmonis	2	23
Neisseria meningitidis serogroup A strain Z2491	2	19
Neisseria meningitidis serogroup B strain MC58	6	163
Nostoc sp. PCC 7120	9	105
Pasteurella multocida Pm70	2	32
Pseudomonas aeruginosa PAO1	3	49
Pyrobaculum aerophilum	0	0
Pyrococcus abyssi GE5	1	20
Pyrococcus horikoshii OT3	1	8
Ralstonia solanacearum	9	121
Rickettsia conorii Malish 7	0	0
Rickettsia prowazekii MadridE	0	0
Salmonella typhimurium LT2	15	314
Sinorhizobium meliloti 1021	5	129
Staphylococcus aureus subsp. Aureus N315	9	113
Streptococcus pneumoniae TIGR4	4	48
Streptococcus pyogenes	3	50
Sulfolobus solfataricus	26	329
Synechocystis sp PCC6803	14	201
Thermoplasma acidophilum	1	11
Thermoplasma volcanium	8	146
Thermotoga maritima MSB8	0	0
Treponema pallidum Nichols	1	16
Ureaplasma urealyticum serovar3	1	27
Vibrio cholerae chromosome I	5	111
Vibrio cholerae chromosome II	5	136
Xylella fastidiosa 9a5c	2	95
Yersinia pestis CO92	21	274
Total	354	6292

DIMOB Islands (ORFs < 300bps excluded)

Organism Name	Num of Islands	Num of Genes in Islands
Aeropyrum pernix K1	0	0
Agrobacterium tumefaciens C58 Cereon Circular	1	50
Agrobacterium tumefaciens C58 Cereon Linear	3	63
Aquifex aeolicus VF5	1	21
Archaeoglobus fulgidus DSM4304	3	28
Bacillus halodurans C125	9	185
Bacillus subtilis 168	3	193
Borrelia burgdorferi B31	0	0
Brucella melitensis choromosome I	6	101
Brucella melitensis choromosome II	1	25
Buchnera sp. APS	0	0
Campylobacter jejuni NCTC11168	1	9
Caulobacter crescentus	7	80
Chlamydia trachomatis D	1	12
Chlamydophila pneumoniae CWL029	2	19
Clostridium acetobutylicum ATCC824	4	71
Corynebacterium glutamicum	5	114
Deinococcus radiodurans chrom 1	5	67
Deinococcus radiodurans chrom 2	1	17
Escherichia coli K12	15	253
Escherichia coli O157	20	432
Escherichia coli O157_EDL933	23	443
Fusobacterium nucleatum subsp. Nucleatum ATCC25586	1	9
Haemophilus influenzae Rd-KW20	1	11
Halobacterium sp. NRC1	6	115
Helicobacter pylori 26695	3	57
Helicobacter pylori J99	1	35
Lactococcus lactis	10	121
Listeria innocua Clip11262	5	97
Mesorhizobium loti	18	286
Methanobacterium thermoautotrophicum deltaH	1	8
Methanococcus jannaschii DSM2661	2	23
Methanopyrus kandleri AV19	0	0
Methanosarcina acetivorans str. C2A	17	220

Mycobacterium leprae	1	10
Mycobacterium tuberculosis CDC1551	8	99
Mycobacterium tuberculosis H37Rv	6	77
Mycoplasma genitalium G37	0	0
Mycoplasma pneumoniae M129	1	8
Mycoplasma pulmonis	3	33
Neisseria meningitidis serogroup A strain Z2491	4	48
Neisseria meningitidis serogroup B strain MC58	5	97
Nostoc sp. PCC 7120	9	100
Pasteurella multocida Pm70	2	30
Pseudomonas aeruginosa PAO1	4	52
Pyrobaculum aerophilum	0	0
Pyrococcus abyssi GE5	1	19
Pyrococcus horikoshii OT3	1	8
Ralstonia solanacearum	10	152
Rickettsia conorii Malish 7	2	36
Rickettsia prowazekii MadridE	0	0
Salmonella typhimurium LT2	11	228
Sinorhizobium meliloti 1021	5	130
Staphylococcus aureus subsp. Aureus N315	6	88
Streptococcus pneumoniae TIGR4	3	32
Streptococcus pyogenes	4	50
Sulfolobus solfataricus	27	321
Synechocystis sp PCC6803	14	190
Thermoplasma acidophilum	1	35
Thermoplasma volcanium	7	125
Thermotoga maritima MSB8	0	0
Treponema pallidum Nichols	1	15
Ureaplasma urealyticum serovar3	1	24
Vibrio cholerae chromosome I	6	120
Vibrio cholerae chromosome II	5	99
Xylella fastidiosa 9a5c	4	112
Yersinia pestis CO92	19	245
Total	347	5748
