# Supplementary material for: Evidence of a Large Novel Gene Pool Associated with Prokaryotic Genomic Islands
Source: PLoS Genet. 2005 Nov 18;1(5):e62. doi: 10.1371/journal.pgen.0010062 (PMC1285063; doi:10.1371/journal.pgen.0010062)
Supplement: Supplementary file 2 [file pgen.0010062.st006.pdf]

Table S6: Summary of Gene Counts in Islands and Outside of Islands (all ORFs included)

Headings: Novel_Island= number of novel genes in islands; Total_Island= number of genes in islands; Novel_Outside= number of nove lgenes outside of islands; Total_Outside= number of genes outside of islands

DINUC Islands, COG Novel Genes

ORG	Novel_Island	Total_Island	Novel_Outside	Total_Outside
Aeropyrum pernix K1	164	326	448	1515
Agrobacterium tumefaciens C58 Cereon Circular	92	328	451	2393
Agrobacterium tumefaciens C58 Cereon Linear	88	165	712	1668
Aquifex aeolicus VF5	28	193	170	1336
Archaeoglobus fulgidus DSM4304	134	339	444	2081
Bacillus halodurans C125	154	429	1049	3637
Bacillus subtilis 168	304	545	944	3567
Borrelia burgdorferi B31	32	146	186	705
Brucella melitensis choromosome I	88	207	297	1852
Brucella melitensis choromosome II	48	127	265	1012
Buchnera sp. APS	4	88	23	476
Campylobacter jejuni NCTC11168	46	170	278	1464
Caulobacter crescentus	200	568	921	3169
Chlamydia trachomatis D	55	145	196	750
Chlamydophila pneumoniae CWL029	55	124	333	930
Clostridium acetobutylicum ATCC824	289	565	892	3107
Corynebacterium glutamicum	239	410	865	2583
Deinococcus radiodurans chrom 1	118	308	646	2321
Deinococcus radiodurans chrom 2	32	54	98	314
Escherichia coli K12	208	549	744	3730
Escherichia coli O157	568	909	1203	4452
Escherichia coli O157_EDL933	489	866	1234	4458
Fusobacterium nucleatum subsp. Nucleatum ATCC25586	97	287	455	1780
Haemophilus influenzae Rd-KW20	32	178	204	1479
Halobacterium sp. NRC1	126	305	464	1770
Helicobacter pylori 26695	69	110	385	1466
Helicobacter pylori J99	66	124	325	1367
Lactococcus lactis	170	310	510	2011
Listeria innocua Clip11262	244	448	463	2520

Mesorhizobium loti	471	903	2260	5843
Methanobacterium thermoautotrophicum deltaH	87	217	320	1656
Methanococcus jannaschii DSM2661	73	269	261	1460
Methanopyrus kandleri AV19	117	304	335	1383
Methanosarcina acetivorans str. C2A	363	699	1568	3841
Mycobacterium leprae	49	160	395	1445
Mycobacterium tuberculosis CDC1551	297	487	1569	3700
Mycobacterium tuberculosis H37Rv	243	499	1315	3428
Mycoplasma genitalium G37	9	62	90	422
Mycoplasma pneumoniae M129	56	102	205	587
Mycoplasma pulmonis	47	111	221	671
Neisseria meningitidis serogroup A strain Z2491	135	242	385	1823
Neisseria meningitidis serogroup B strain MC58	176	286	362	1793
Nostoc sp. PCC 7120	373	760	2105	4606
Pasteurella multocida Pm70	48	203	146	1812
Pseudomonas aeruginosa PAO1	206	690	1465	4877
Pyrobaculum aerophilum	236	348	890	2257
Pyrococcus abyssi GE5	51	259	243	1510
Pyrococcus horikoshii OT3	81	202	320	1599
Ralstonia solanacearum	188	471	711	2969
Rickettsia conorii Malish 7	100	198	397	1176
Rickettsia prowazekii MadridE	26	127	89	708
Salmonella typhimurium LT2	270	615	781	3836
Sinorhizobium meliloti 1021	223	443	931	2898
Staphylococcus aureus subsp. Aureus N315	162	320	439	2273
Streptococcus pneumoniae TIGR4	58	152	490	1942
Streptococcus pyogenes	81	206	265	1491
Sulfolobus solfataricus	269	465	876	2512
Synechocystis sp PCC6803	153	339	905	2828
Thermoplasma acidophilum	63	231	222	1251
Thermoplasma volcanium	66	246	228	1253
Thermotoga maritima MSB8	63	237	272	1621
Treponema pallidum Nichols	51	150	260	886
Ureaplasma urealyticum serovar3	40	78	159	536
Vibrio cholerae chromosome I	81	304	533	2438
Vibrio cholerae chromosome II	122	201	372	892

Xylella fastidiosa 9a5c	214	375	875	2391
Yersinia pestis CO92	167	459	812	3426

DIMOB Islands, COG Novel Genes

ORG	Novel_Island	Total_Island	Novel_Outside	Total_Outside
Agrobacterium tumefaciens C58 Cereon Circular	27	50	516	2671
Agrobacterium tumefaciens C58 Cereon Linear	42	71	758	1762
Aquifex aeolicus VF5	1	17	197	1512
Archaeoglobus fulgidus DSM4304	7	21	571	2399
Bacillus halodurans C125	80	201	1123	3865
Bacillus subtilis 168	203	267	1045	3845
Brucella melitensis choromosome I	56	112	329	1947
Brucella melitensis choromosome II	25	46	288	1093
Caulobacter crescentus	33	61	1088	3676
Chlamydia trachomatis D	5	11	246	884
Chlamydophila pneumoniae CWL029	5	8	383	1046
Clostridium acetobutylicum ATCC824	62	85	1119	3587
Corynebacterium glutamicum	96	154	1008	2839
Deinococcus radiodurans chrom 1	36	56	728	2573
Deinococcus radiodurans chrom 2	18	23	112	345
Escherichia coli K12	142	275	810	4004
Escherichia coli O157	295	415	1476	4946
Escherichia coli O157_EDL933	321	486	1402	4838
Fusobacterium nucleatum subsp. Nucleatum ATCC25586	20	36	532	2031
Haemophilus influenzae Rd-KW20	6	12	230	1645
Halobacterium sp. NRC1	68	138	522	1937
Helicobacter pylori 26695	35	62	419	1514
Helicobacter pylori J99	28	50	363	1441
Lactococcus lactis	98	179	582	2142
Listeria innocua Clip11262	74	100	633	2868
Mesorhizobium loti	203	344	2528	6402
Methanococcus jannaschii DSM2661	7	25	327	1704
Methanosarcina acetivorans str. C2A	122	244	1809	4296
Mycobacterium tuberculosis CDC1551	59	91	1807	4096
Mycobacterium tuberculosis H37Rv	55	104	1503	3823
Mycoplasma pneumoniae M129	4	8	257	681

Mycoplasma pulmonis	12	23	256	759
Neisseria meningitidis serogroup A strain Z2491	14	19	506	2046
Neisseria meningitidis serogroup B strain MC58	82	163	456	1916
Nostoc sp. PCC 7120	61	105	2417	5261
Pasteurella multocida Pm70	11	32	183	1983
Pseudomonas aeruginosa PAO1	20	49	1651	5518
Pyrococcus abyssi GE5	6	20	288	1749
Pyrococcus horikoshii OT3	5	8	396	1793
Ralstonia solanacearum	69	121	830	3319
Salmonella typhimurium LT2	172	314	879	4137
Sinorhizobium meliloti 1021	89	129	1065	3212
Staphylococcus aureus subsp. Aureus N315	51	113	550	2480
Streptococcus pneumoniae TIGR4	31	48	517	2046
Streptococcus pyogenes	23	50	323	1647
Sulfolobus solfataricus	225	329	920	2648
Synechocystis sp PCC6803	102	201	956	2966
Thermoplasma acidophilum	3	11	282	1471
Thermoplasma volcanium	54	146	240	1353
Treponema pallidum Nichols	4	16	307	1020
Ureaplasma urealyticum serovar3	26	27	173	587
Vibrio cholerae chromosome I	49	111	565	2631
Vibrio cholerae chromosome II	77	136	417	957
Xylella fastidiosa 9a5c	47	95	1042	2671
Yersinia pestis CO92	100	274	879	3611

DINUC Islands, SUPERFAMILY Novel Genes

ORG	Novel_Island	Total_Island	Novel_Outside	Total_Outside
Aeropyrum pernix K1	176	326	945	1515
Agrobacterium tumefaciens C58 Cereon Circular	147	328	1102	2343
Agrobacterium tumefaciens C58 Cereon Linear	72	165	600	1597
Aquifex aeolicus VF5	75	193	543	1319
Archaeoglobus fulgidus DSM4304	183	339	998	2060
Bacillus halodurans C125	191	429	1659	3436
Bacillus subtilis 168	304	545	1607	3300
Borrelia burgdorferi B31	62	146	416	705
Brucella melitensis choromosome I	88	207	736	1740

Brucella melitensis choromosome II	74	127	399	966
Buchnera sp. APS	20	88	174	476
Campylobacter jejuni NCTC11168	66	170	694	1464
Caulobacter crescentus	247	568	1574	3108
Chlamydia trachomatis D	69	145	424	739
Chlamydophila pneumoniae CWL029	53	124	536	922
Clostridium acetobutylicum ATCC824	284	565	1656	3022
Corynebacterium glutamicum	224	410	1205	2429
Deinococcus radiodurans chrom 1	130	308	1112	2265
Deinococcus radiodurans chrom 2	29	54	127	291
Escherichia coli K12	266	549	1777	3455
Escherichia coli O157	553	909	2203	4037
Escherichia coli O157_EDL933	493	866	2123	3972
Fusobacterium nucleatum subsp. Nucleatum ATCC25586	122	287	920	1744
Haemophilus influenzae Rd-KW20	66	178	625	1467
Halobacterium sp. NRC1	152	305	811	1632
Helicobacter pylori 26695	80	110	660	1404
Helicobacter pylori J99	84	124	607	1317
Lactococcus lactis	165	310	835	1832
Listeria innocua Clip11262	256	448	1177	2420
Mesorhizobium loti	419	903	2661	5499
Methanobacterium thermoautotrophicum deltaH	121	217	761	1656
Methanococcus jannaschii DSM2661	109	269	735	1435
Methanopyrus kandleri AV19	156	304	773	1383
Methanosarcina acetivorans str. C2A	304	699	2015	3597
Mycobacterium leprae	60	160	656	1445
Mycobacterium tuberculosis CDC1551	299	487	1891	3609
Mycobacterium tuberculosis H37Rv	234	499	1629	3324
Mycoplasma genitalium G37	16	62	196	422
Mycoplasma pneumoniae M129	51	102	335	579
Mycoplasma pulmonis	54	111	360	648
Neisseria meningitidis serogroup A strain Z2491	136	242	940	1804
Neisseria meningitidis serogroup B strain MC58	181	286	800	1630
Nostoc sp. PCC 7120	359	760	2559	4501
Pasteurella multocida Pm70	80	203	743	1780
Pseudomonas aeruginosa PAO1	284	690	2201	4828

Pyrobaculum aerophilum	222	348	1357	2257
Pyrococcus abyssi GE5	112	259	739	1490
Pyrococcus horikoshii OT3	112	202	782	1591
Ralstonia solanacearum	214	471	1374	2848
Rickettsia conorii Malish 7	121	198	772	1176
Rickettsia prowazekii MadridE	49	127	355	708
Salmonella typhimurium LT2	298	615	1676	3522
Sinorhizobium meliloti 1021	168	443	1269	2769
Staphylococcus aureus subsp. Aureus N315	162	320	1040	2160
Streptococcus pneumoniae TIGR4	65	152	845	1894
Streptococcus pyogenes	96	206	655	1441
Sulfolobus solfataricus	250	465	1097	2183
Synechocystis sp PCC6803	142	339	1175	2627
Thermoplasma acidophilum	86	231	633	1240
Thermoplasma volcanium	106	246	495	1107
Thermotoga maritima MSB8	90	237	748	1621
Treponema pallidum Nichols	69	150	507	870
Ureaplasma urealyticum serovar3	37	78	259	509
Vibrio cholerae chromosome I	106	304	1133	2327
Vibrio cholerae chromosome II	141	201	463	756
Xylella fastidiosa 9a5c	232	375	1453	2296
Yersinia pestis CO92	217	459	1518	3152

DIMOB Islands, SUPERFAMILY Novel Genes

ORG	Novel_Island	Total_Island	Novel_Outside	Total_Outside
Agrobacterium tumefaciens C58 Cereon Circular	32	50	939	2671
Agrobacterium tumefaciens C58 Cereon Linear	28	71	550	1762
Aquifex aeolicus VF5	4	17	438	1512
Archaeoglobus fulgidus DSM4304	14	21	849	2399
Bacillus halodurans C125	88	201	1534	3865
Bacillus subtilis 168	186	267	1447	3845
Brucella melitensis choromosome I	59	112	670	1947
Brucella melitensis choromosome II	32	46	360	1093
Caulobacter crescentus	34	61	1280	3676
Chlamydia trachomatis D	7	11	352	884
Chlamydophila pneumoniae CWL029	4	8	469	1046

Clostridium acetobutylicum ATCC824	52	85	1408	3587
Corynebacterium glutamicum	86	154	1087	2839
Deinococcus radiodurans chrom 1	31	56	959	2573
Deinococcus radiodurans chrom 2	15	23	110	345
Escherichia coli K12	159	275	1610	4004
Escherichia coli O157	263	415	1999	4946
Escherichia coli O157_EDL933	300	486	1936	4838
Fusobacterium nucleatum subsp. Nucleatum ATCC25586	19	36	772	2031
Haemophilus influenzae Rd-KW20	6	12	519	1645
Halobacterium sp. NRC1	74	138	722	1937
Helicobacter pylori 26695	43	62	649	1514
Helicobacter pylori J99	38	50	579	1441
Lactococcus lactis	86	179	783	2142
Listeria innocua Clip11262	59	100	1026	2868
Mesorhizobium loti	175	344	2346	6402
Methanococcus jannaschii DSM2661	9	25	591	1704
Methanosarcina acetivorans str. C2A	99	244	1765	4296
Mycobacterium tuberculosis CDC1551	67	91	1727	4096
Mycobacterium tuberculosis H37Rv	56	104	1412	3823
Mycoplasma pneumoniae M129	5	8	287	681
Mycoplasma pulmonis	11	23	315	759
Neisseria meningitidis serogroup A strain Z2491	14	19	839	2046
Neisseria meningitidis serogroup B strain MC58	100	163	758	1916
Nostoc sp. PCC 7120	52	105	2211	5261
Pasteurella multocida Pm70	17	32	635	1983
Pseudomonas aeruginosa PAO1	23	49	1821	5518
Pyrococcus abyssi GE5	8	20	604	1749
Pyrococcus horikoshii OT3	2	8	698	1793
Ralstonia solanacearum	60	121	1178	3319
Salmonella typhimurium LT2	186	314	1487	4137
Sinorhizobium meliloti 1021	56	129	1067	3212
Staphylococcus aureus subsp. Aureus N315	52	113	943	2480
Streptococcus pneumoniae TIGR4	32	48	774	2046
Streptococcus pyogenes	28	50	567	1647
Sulfolobus solfataricus	190	329	1021	2648
Synechocystis sp PCC6803	85	201	1094	2966

Thermoplasma acidophilum	4	11	495	1471
Thermoplasma volcanium	71	146	430	1353
Treponema pallidum Nichols	6	16	436	1020
Ureaplasma urealyticum serovar3	23	27	222	587
Vibrio cholerae chromosome I	46	111	1000	2631
Vibrio cholerae chromosome II	93	136	446	957
Xylella fastidiosa 9a5c	46	95	1359	2671
Yersinia pestis CO92	133	274	1417	3611
