# Supplementary material for: Evidence of a Large Novel Gene Pool Associated with Prokaryotic Genomic Islands
Source: PLoS Genet. 2005 Nov 18;1(5):e62. doi: 10.1371/journal.pgen.0010062 (PMC1285063; doi:10.1371/journal.pgen.0010062)
Supplement: Supplementary file 3 [file pgen.0010062.st007.pdf]

Table S7: Gene count in each of the COG categories (excluding the COG-None Categories)

DINUC Islands (all ORFs included)

ORG	A_OUTSIDE	A_ISLAND	B_OUTSIDE	B_ISLAND	C_OUTSIDE	C_ISLAND	D_OUTSIDE	D_ISLAND	E_OUTSIDE	E_ISLAND	F_OUTSIDE	F_ISLAND	G_OUTSIDE	G_ISLAND	H_OUTSIDE
Aeropyrum pernix K1	1	0	2	0	100	4	3	1	116	21	33	3	54	4	53
Agrobacterium tumefaciens C58 Cereon Circular	0	0	1	0	105	19	16	0	208	16	65	4	107	7	76
Agrobacterium tumefaciens C58 Cereon Linear	0	0	0	0	57	2	10	0	158	2	16	2	100	4	38
Aquifex aeolicus VF5	1	0	2	0	76	41	16	0	95	14	41	4	38	4	80
Archaeoglobus fulgidus DSM4304	1	0	5	1	189	25	13	1	130	13	44	4	43	5	89
Bacillus halodurans C125	0	0	1	0	157	2	32	1	262	8	69	3	221	12	104
Bacillus subtilis 168	0	0	1	0	159	6	33	1	270	6	70	9	216	14	104
Borrelia burgdorferi B31	0	0	0	0	18	5	11	3	21	5	18	2	39	4	9
Brucella melitensis choromosome I	0	0	1	0	87	13	18	1	164	5	46	2	70	3	87
Brucella melitensis choromosome II	0	0	0	0	61	4	7	0	140	3	18	0	71	11	26
Buchnera sp. APS	1	0	0	0	40	2	11	0	53	1	18	7	31	0	30
Campylobacter jejuni NCTC11168	0	0	0	0	82	14	15	1	125	7	42	2	33	7	67
Caulobacter crescentus	0	0	2	0	134	22	21	1	195	19	57	8	122	9	83
Chlamydia trachomatis D	0	0	2	0	38	3	9	0	44	7	16	0	33	2	34
Chlamydophila pneumoniae CWL029	0	0	2	0	40	0	8	1	48	7	22	0	32	3	40
Clostridium acetobutylicum ATCC824	0	0	0	0	100	12	29	5	207	9	65	5	185	19	91
Corynebacterium glutamicum	1	0	0	0	93	12	14	3	176	2	60	2	116	6	94
Deinococcus radiodurans chrom 1	0	0	1	0	89	23	17	2	176	19	59	7	82	3	57
Deinococcus radiodurans chrom 2	0	0	0	0	20	0	1	1	32	0	7	0	9	1	17
Escherichia coli K12	2	0	0	0	250	22	33	0	312	13	79	6	292	7	114
Escherichia coli O157	2	0	0	0	265	7	32	0	321	6	79	6	288	3	126
Escherichia coli O157_EDL933	2	0	0	0	263	7	32	0	321	6	79	6	292	3	127
Fusobacterium nucleatum subsp. Nucleatum ATCC25586	0	0	0	0	92	9	20	0	131	21	38	2	80	11	79
Haemophilus influenzae Rd-KW20	1	0	0	0	83	7	18	2	128	7	44	4	81	15	64
Halobacterium sp. NRC1	1	0	2	0	98	15	9	2	124	10	49	6	35	1	74
Helicobacter pylori 26695	0	0	0	0	68	0	19	1	87	0	34	0	36	0	65
Helicobacter pylori J99	0	0	0	0	66	1	17	3	84	3	32	2	36	0	66
Lactococcus lactis	0	0	0	0	58	5	17	0	153	2	69	2	130	5	53
Listeria innocua Clip11262	0	0	0	0	90	8	26	3	185	17	66	1	211	12	85
Mesorhizobium loti	0	0	5	0	220	21	22	2	423	38	76	7	230	12	141
Methanobacterium thermoautotrophicum deltaH	1	0	4	0	141	10	10	0	97	1	49	0	37	2	87
Methanococcus jannaschii DSM2661	1	0	4	1	104	29	16	1	92	9	41	4	42	2	80
Methanopyrus kandleri AV19	1	0	4	0	95	15	15	2	90	12	41	2	29	3	72
Methanosarcina acetivorans str. C2A	1	0	2	0	203	35	18	2	178	27	52	10	86	5	117
Mycobacterium leprae	1	0	0	0	55	10	18	3	117	4	46	3	61	3	65
Mycobacterium tuberculosis CDC1551	1	0	0	0	156	10	34	5	179	4	60	3	102	9	113
Mycobacterium tuberculosis H37Rv	1	0	0	0	154	19	36	4	184	7	61	4	102	9	107
Mycoplasma genitalium G37	0	0	0	0	19	1	5	0	14	1	18	3	23	3	9
Mycoplasma pneumoniae M129	0	0	0	0	19	1	5	0	19	5	21	0	32	5	12
Mycoplasma pulmonis	0	0	0	0	29	0	5	0	25	0	24	0	50	12	12
Neisseria meningitidis serogroup A strain Z2491	1	0	1	0	102	3	22	3	134	2	42	2	55	2	73
Neisseria meningitidis serogroup B strain MC58	1	0	1	0	104	3	22	3	136	0	41	2	55	1	73
Nostoc sp. PCC 7120	1	0	1	1	157	23	25	3	190	22	58	5	122	13	125
Pasteurella multocida Pm70	1	0	0	0	111	2	22	2	153	3	57	0	151	8	85
Pseudomonas aeruginosa PAO1	2	0	3	0	240	35	26	6	386	29	86	13	134	10	141
Pyrobaculum aerophilum	1	0	2	0	138	10	7	0	136	6	45	4	68	3	79
Pyrococcus abyssi GE5	1	0	3	0	100	19	15	2	109	18	44	3	71	5	56
Pyrococcus horikoshii OT3	1	0	3	0	86	13	16	1	96	8	42	3	78	2	52
Ralstonia solanacearum	2	0	2	0	173	3	22	1	220	22	70	2	96	21	97
Rickettsia conorii Malish 7	0	0	0	0	73	4	16	2	32	0	20	2	18	2	25
Rickettsia prowazekii MadridE	0	0	0	0	56	16	13	1	24	1	15	2	15	4	25
Salmonella typhimurium LT2	2	0	0	0	243	15	32	1	321	6	79	2	285	18	145
Sinorhizobium meliloti 1021	0	0	1	0	115	18	23	1	217	11	68	3	87	7	95
Staphylococcus aureus subsp. Aureus N315	0	0	1	0	101	2	22	1	205	7	64	2	118	4	77
Streptococcus pneumoniae TIGR4	0	0	0	0	46	0	20	0	133	3	58	2	169	9	43
Streptococcus pyogenes	0	0	0	0	56	1	14	2	86	10	56	3	118	4	34
Sulfolobus solfataricus	1	0	2	1	145	26	9	0	179	10	53	7	82	14	80
Synechocystis sp PCC6803	0	0	1	0	128	7	22	2	157	7	56	3	95	10	105
Thermoplasma acidophilum	0	0	0	0	100	9	6	1	78	35	44	2	65	5	60
Thermoplasma volcanium	0	0	0	0	102	10	7	1	100	5	44	0	55	8	64
Thermotoga maritima MSB8	0	0	0	0	98	12	17	1	126	24	46	4	125	30	44
Treponema pallidum Nichols	0	0	0	0	35	0	12	1	22	2	21	1	37	4	16
Ureaplasma urealyticum serovar3	0	0	0	0	16	0	4	0	20	0	21	0	15	0	9
Vibrio cholerae chromosome I	1	0	1	0	110	21	29	1	181	4	55	5	95	12	107
Vibrio cholerae chromosome II	0	0	0	0	37	0	2	3	50	6	10	2	52	0	14
Xylella fastidiosa 9a5c	1	0	0	0	81	5	25	2	108	8	36	4	68	8	67
Yersinia pestis CO92	1	0	0	0	162	12	31	0	272	5	67	2	220	16	105

Table S7: Gene count in each of the COG categories (excluding the COG-None Categories)

DINUC Islands (all ORFs included)

ORG	H_ISLAND	I_OUTSIDE	I_ISLAND	J_OUTSIDE	J_ISLAND	K_OUTSIDE	K_ISLAND	L_OUTSIDE	L_ISLAND	M_OUTSIDE	M_ISLAND	N_OUTSIDE	N_ISLAND	O_OUTSIDE	O_ISLAND
Aeropyrum pernix K1	3	34	3	130	26	66	4	49	13	13	10	10	1	43	12
Agrobacterium tumefaciens C58 Cereon Circular	9	75	4	100	37	130	17	81	8	129	13	18	26	88	14
Agrobacterium tumefaciens C58 Cereon Linear	3	33	3	14	4	50	1	26	13	54	7	3	0	17	3
Aquifex aeolicus VF5	2	41	3	129	7	37	6	68	11	89	18	37	7	64	7
Archaeoglobus fulgidus DSM4304	2	73	5	135	22	93	7	83	13	35	12	17	2	59	8
Bacillus halodurans C125	4	91	6	103	51	192	28	153	31	112	16	40	13	90	4
Bacillus subtilis 168	2	90	4	114	42	183	26	106	25	166	7	49	6	87	8
Borrelia burgdorferi B31	3	15	1	84	35	17	3	45	2	46	6	36	11	24	6
Brucella melitensis choromosome I	0	74	0	95	37	85	9	85	16	108	9	2	1	96	4
Brucella melitensis choromosome II	1	20	1	24	1	48	5	16	5	37	9	18	12	19	2
Buchnera sp. APS	2	12	2	73	39	16	1	31	7	23	3	10	12	34	0
Campylobacter jejuni NCTC11168	6	36	1	108	17	33	4	60	2	98	21	47	3	68	2
Caulobacter crescentus	12	107	13	120	40	151	24	113	18	168	20	33	29	103	18
Chlamydia trachomatis D	2	32	3	85	28	20	1	44	9	31	7	14	0	31	4
Chlamydophila pneumoniae CWL029	0	27	3	85	31	19	2	50	3	32	5	14	0	34	1
Clostridium acetobutylicum ATCC824	14	68	2	110	49	163	32	123	12	144	16	49	19	79	4
Corynebacterium glutamicum	3	60	6	105	28	128	12	94	15	73	16	2	0	72	10
Deinococcus radiodurans chrom 1	9	75	4	137	12	91	8	92	14	70	9	9	2	86	5
Deinococcus radiodurans chrom 2	0	11	0	2	0	9	1	4	1	7	9	4	0	13	1
Escherichia coli K12	12	88	3	116	53	173	26	150	45	200	34	76	6	120	8
Escherichia coli O157	4	95	1	128	41	185	25	169	50	213	25	76	11	131	8
Escherichia coli O157_EDL933	4	90	6	128	42	185	27	164	56	208	34	77	10	133	10
Fusobacterium nucleatum subsp. Nucleatum ATCC25586	2	43	9	102	35	69	9	95	26	85	18	4	2	44	7
Haemophilus influenzae Rd-KW20	1	36	5	106	34	60	7	88	5	103	5	6	0	74	3
Halobacterium sp. NRC1	9	54	1	116	26	83	9	76	22	44	8	23	10	63	4
Helicobacter pylori 26695	0	42	0	119	0	23	0	83	13	96	0	49	1	62	0
Helicobacter pylori J99	0	39	2	117	2	23	1	75	10	90	5	44	3	62	1
Lactococcus lactis	2	49	0	105	32	111	14	104	15	79	16	6	2	44	2
Listeria innocua Clip11262	5	50	4	109	36	158	23	108	18	90	6	35	7	59	1
Mesorhizobium loti	20	144	18	136	35	212	26	148	51	220	15	44	7	154	19
Methanobacterium thermoautotrophicum deltaH	19	25	0	113	27	64	4	63	8	45	6	2	1	56	5
Methanococcus jannaschii DSM2661	14	15	0	110	40	48	11	77	9	25	9	10	9	42	4
Methanopyrus kandleri AV19	17	15	2	139	22	42	7	49	15	22	4	3	0	36	6
Methanosarcina acetivorans str. C2A	19	34	4	151	10	103	24	167	36	77	8	42	0	110	9
Mycobacterium leprae	4	73	6	91	28	49	10	57	5	60	4	4	1	53	8
Mycobacterium tuberculosis CDC1551	8	120	11	126	16	125	10	139	17	92	10	5	3	85	10
Mycobacterium tuberculosis H37Rv	12	118	13	119	22	125	10	147	24	91	15	4	5	81	15
Mycoplasma genitalium G37	4	8	1	75	25	13	1	34	6	12	0	0	0	20	0
Mycoplasma pneumoniae M129	1	8	1	85	16	14	0	39	4	11	1	0	0	20	0
Mycoplasma pulmonis	0	5	3	82	21	16	5	51	11	8	0	0	0	16	2
Neisseria meningitidis serogroup A strain Z2491	1	43	0	106	41	60	5	133	8	122	7	22	5	66	2
Neisseria meningitidis serogroup B strain MC58	0	50	0	111	40	59	5	117	16	117	8	26	0	67	2
Nostoc sp. PCC 7120	11	62	6	112	46	87	18	138	16	176	12	25	3	138	22
Pasteurella multocida Pm70	4	41	1	112	40	74	11	89	14	128	13	7	1	87	3
Pseudomonas aeruginosa PAO1	12	148	13	128	51	177	29	116	21	183	28	112	17	145	29
Pyrobaculum aerophilum	3	39	4	139	6	79	5	65	11	27	7	6	0	61	9
Pyrococcus abyssi GE5	3	17	2	131	21	70	15	56	8	42	7	15	12	45	4
Pyrococcus horikoshii OT3	4	18	0	148	7	79	4	75	2	33	10	13	15	44	5
Ralstonia solanacearum	10	94	8	115	37	121	27	134	24	144	10	38	17	118	6
Rickettsia conorii Malish 7	6	40	1	99	27	24	1	60	11	78	5	2	2	49	9
Rickettsia prowazekii MadridE	0	28	0	85	39	17	3	54	3	63	10	2	0	45	7
Salmonella typhimurium LT2	3	81	3	123	46	179	30	134	26	202	37	75	11	124	26
Sinorhizobium meliloti 1021	10	75	4	117	36	87	9	101	14	106	7	31	17	100	10
Staphylococcus aureus subsp. Aureus N315	3	66	0	107	42	120	9	116	25	100	5	4	1	68	3
Streptococcus pneumoniae TIGR4	1	33	0	111	32	110	6	109	8	77	10	5	0	53	1
Streptococcus pyogenes	0	48	1	103	37	91	13	100	9	63	3	6	4	49	1
Sulfolobus solfataricus	1	70	3	133	21	95	7	121	23	54	8	12	2	70	4
Synechocystis sp PCC6803	4	51	1	114	29	60	8	96	20	128	12	23	7	118	6
Thermoplasma acidophilum	10	50	1	99	33	68	10	56	13	29	10	13	0	42	4
Thermoplasma volcanium	3	43	5	105	27	66	6	57	52	32	8	11	2	40	9
Thermotoga maritima MSB8	4	27	3	121	12	65	9	80	7	51	13	52	5	49	2
Treponema pallidum Nichols	4	18	0	86	32	22	5	52	7	60	4	36	8	39	4
Ureaplasma urealyticum serovar3	0	8	0	78	25	17	2	46	5	6	0	0	0	18	1
Vibrio cholerae chromosome I	6	59	4	109	47	102	23	101	21	125	12	80	10	95	7
Vibrio cholerae chromosome II	0	13	1	8	8	32	4	16	16	20	5	6	0	23	3
Xylella fastidiosa 9a5c	7	48	4	111	23	67	16	110	12	131	7	52	0	79	12
Yersinia pestis CO92	9	74	1	110	55	143	22	130	17	177	17	89	23	110	10

Table S7: Gene count in each of the COG categories (excluding the COG-None Categories)

DINUC Islands (all ORFs included)

ORG	P_OUTSIDE	P_ISLAND	Q_OUTSIDE	Q_ISLAND	R_OUTSIDE	R_ISLAND	S_OUTSIDE	S_ISLAND	T_OUTSIDE	T_ISLAND	U_OUTSIDE	U_ISLAND	V_OUTSIDE	V_ISLAND
Aeropyrum pernix K1	40	6	7	3	177	26	101	16	11	1	9	2	15	3
Agrobacterium tumefaciens C58 Cereon Circular	120	6	27	2	240	16	229	35	76	1	26	2	25	0
Agrobacterium tumefaciens C58 Cereon Linear	74	0	24	2	123	19	116	11	26	0	5	1	12	0
Aquifex aeolicus VF5	50	6	14	4	136	20	87	8	36	2	21	0	8	1
Archaeoglobus fulgidus DSM4304	74	6	15	2	258	30	211	37	40	5	12	3	18	2
Bacillus halodurans C125	142	12	35	3	312	19	307	45	99	9	17	4	49	4
Bacillus subtilis 168	153	6	48	3	312	25	303	38	93	3	20	5	46	5
Borrelia burgdorferi B31	16	1	0	0	56	14	34	9	12	1	10	2	8	1
Brucella melitensis choromosome I	63	2	18	0	179	3	190	9	46	3	20	2	21	0
Brucella melitensis choromosome II	56	1	17	0	80	3	54	10	18	1	2	6	15	4
Buchnera sp. APS	14	1	1	0	23	4	12	3	5	0	11	0	4	0
Campylobacter jejuni NCTC11168	76	5	16	3	120	13	83	10	29	2	23	3	25	1
Caulobacter crescentus	97	17	58	8	263	26	280	54	69	12	32	18	40	0
Chlamydia trachomatis D	15	3	2	0	48	10	26	7	10	2	17	2	3	0
Chlamydophila pneumoniae CWL029	22	0	1	2	52	6	39	1	10	2	17	2	3	0
Clostridium acetobutylicum ATCC824	93	5	19	1	287	26	240	34	94	5	11	5	58	2
Corynebacterium glutamicum	137	12	30	2	209	17	162	14	47	8	15	1	30	2
Deinococcus radiodurans chrom 1	75	6	23	5	225	23	198	25	64	6	15	3	34	5
Deinococcus radiodurans chrom 2	17	2	9	0	18	6	13	0	12	0	0	0	11	0
Escherichia coli K12	168	22	41	5	313	34	287	35	96	1	32	5	44	4
Escherichia coli O157	198	12	42	1	362	51	351	62	102	10	33	14	51	4
Escherichia coli O157_EDL933	198	20	43	1	353	56	351	59	97	9	31	17	50	4
Fusobacterium nucleatum subsp. Nucleatum ATCC25586	77	4	8	4	160	16	116	13	23	2	26	0	33	0
Haemophilus influenzae Rd-KW20	58	19	13	0	120	15	125	10	28	4	24	2	15	1
Halobacterium sp. NRC1	62	17	17	1	184	12	134	20	30	2	11	1	17	3
Helicobacter pylori 26695	47	2	8	0	100	4	71	3	14	2	28	13	30	2
Helicobacter pylori J99	47	3	7	1	107	1	63	5	16	0	24	15	27	0
Lactococcus lactis	78	3	15	0	173	12	174	19	34	4	10	3	39	2
Listeria innocua Clip11262	108	3	28	3	246	13	259	31	75	5	15	4	54	4
Mesorhizobium loti	162	15	70	9	455	46	510	70	103	7	59	9	49	5
Methanobacterium thermoautotrophicum deltaH	63	9	6	2	214	19	202	15	30	1	11	1	16	0
Methanococcus jannaschii DSM2661	62	2	8	1	204	20	182	30	9	1	12	0	15	0
Methanopyrus kandleri AV19	28	2	8	1	175	32	161	36	12	2	9	4	2	3
Methanosarcina acetivorans str. C2A	143	20	21	4	339	59	309	47	52	3	18	4	50	10
Mycobacterium leprae	46	0	34	3	93	8	74	4	29	3	11	4	13	0
Mycobacterium tuberculosis CDC1551	106	5	78	5	272	29	209	27	78	3	15	3	36	2
Mycobacterium tuberculosis H37Rv	111	3	79	6	273	33	200	39	73	7	14	4	33	5
Mycoplasma genitalium G37	17	0	0	0	35	6	15	0	3	0	5	1	7	1
Mycoplasma pneumoniae M129	17	0	0	0	44	2	14	3	3	0	7	0	12	7
Mycoplasma pulmonis	16	0	0	0	47	5	25	3	3	0	9	0	27	2
Neisseria meningitidis serogroup A strain Z2491	76	6	14	2	143	6	166	2	24	2	19	6	14	2
Neisseria meningitidis serogroup B strain MC58	78	4	14	4	137	3	161	4	26	2	18	12	17	1
Nostoc sp. PCC 7120	176	34	60	15	327	48	310	54	129	9	27	7	55	19
Pasteurella multocida Pm70	110	9	16	1	173	9	158	16	41	3	24	14	26	1
Pseudomonas aeruginosa PAO1	238	28	82	9	415	51	417	62	130	10	53	17	50	14
Pyrobaculum aerophilum	57	2	11	1	220	22	136	15	19	3	11	0	21	1
Pyrococcus abyssi GE5	55	16	9	3	225	35	161	28	14	1	11	1	17	5
Pyrococcus horikoshii OT3	44	11	13	2	222	18	171	13	16	1	10	2	19	0
Ralstonia solanacearum	98	9	49	7	269	26	247	32	83	3	42	16	24	2
Rickettsia conorii Malish 7	19	3	7	2	85	7	62	7	19	3	30	2	21	2
Rickettsia prowazekii MadridE	15	5	10	0	57	3	45	3	13	1	29	2	8	1
Salmonella typhimurium LT2	168	8	51	1	327	51	319	43	85	8	37	7	43	3
Sinorhizobium meliloti 1021	97	13	28	1	235	24	298	26	32	1	35	2	19	6
Staphylococcus aureus subsp. Aureus N315	123	7	24	4	215	15	204	19	45	3	14	4	40	2
Streptococcus pneumoniae TIGR4	70	7	10	0	151	7	146	4	39	1	16	1	53	2
Streptococcus pyogenes	54	4	7	0	131	7	129	15	36	3	12	2	33	6
Sulfolobus solfataricus	55	4	30	1	261	37	143	22	13	1	9	2	19	2
Synechocystis sp PCC6803	139	12	35	1	242	22	197	16	95	15	25	1	36	3
Thermoplasma acidophilum	35	1	13	3	137	18	103	9	9	0	10	2	12	2
Thermoplasma volcanium	29	4	11	2	129	19	100	11	9	1	10	1	11	6
Thermotoga maritima MSB8	64	5	12	1	170	23	122	9	46	1	11	1	23	8
Treponema pallidum Nichols	20	3	2	0	68	12	42	9	21	1	11	1	6	1
Ureaplasma urealyticum serovar3	25	0	0	0	45	0	26	2	3	0	7	0	13	3
Vibrio cholerae chromosome I	95	6	31	3	191	13	185	16	92	2	28	6	33	4
Vibrio cholerae chromosome II	52	1	7	0	76	15	68	12	22	0	5	0	7	3
Xylella fastidiosa 9a5c	60	4	21	0	177	21	160	19	45	2	40	6	29	1
Yersinia pestis CO92	179	10	38	6	240	19	313	42	76	4	39	18	38	4

Table S7: Gene count in each of the COG categories (excluding the COG-None Categories)

DIMOB Islands (all ORFs included)

ORG	A_OUTSIDE	A_ISLAND	B_OUTSIDE	B_ISLAND	C_OUTSIDE	C_ISLAND	D_OUTSIDE	D_ISLAND	E_OUTSIDE	E_ISLAND	F_OUTSIDE	F_ISLAND	G_OUTSIDE	G_ISLAND	H_OUTSIDE
Aeropyrum pernix K1	1	0	2	0	104	0	4	0	137	0	36	0	58	0	56
Agrobacterium tumefaciens C58 Cereon Circular	0	0	1	0	124	0	16	0	222	2	68	1	113	1	85
Agrobacterium tumefaciens C58 Cereon Linear	0	0	0	0	58	1	10	0	160	0	18	0	103	1	41
Aquifex aeolicus VF5	1	0	2	0	114	3	16	0	103	6	45	0	42	0	82
Archaeoglobus fulgidus DSM4304	1	0	5	1	213	1	14	0	143	0	48	0	48	0	91
Bacillus halodurans C125	0	0	1	0	158	1	33	0	269	1	71	1	232	1	108
Bacillus subtilis 168	0	0	1	0	164	1	34	0	275	1	75	4	228	2	106
Borrelia burgdorferi B31	0	0	0	0	23	0	14	0	26	0	20	0	43	0	12
Brucella melitensis choromosome I	0	0	1	0	92	8	19	0	165	4	47	1	70	3	87
Brucella melitensis choromosome II	0	0	0	0	64	1	7	0	143	0	18	0	81	1	27
Buchnera sp. APS	1	0	0	0	42	0	11	0	54	0	25	0	31	0	32
Campylobacter jejuni NCTC11168	0	0	0	0	96	0	16	0	132	0	44	0	40	0	73
Caulobacter crescentus	0	0	2	0	156	0	22	0	213	1	63	2	130	1	95
Chlamydia trachomatis D	0	0	2	0	41	0	9	0	51	0	16	0	35	0	36
Chlamydophila pneumoniae CWL029	0	0	2	0	40	0	9	0	55	0	22	0	35	0	40
Clostridium acetobutylicum ATCC824	0	0	0	0	112	0	34	0	216	0	70	0	203	1	105
Corynebacterium glutamicum	1	0	0	0	102	3	16	1	178	0	62	0	122	0	97
Deinococcus radiodurans chrom 1	0	0	1	0	112	0	19	0	194	1	66	0	84	1	64
Deinococcus radiodurans chrom 2	0	0	0	0	20	0	2	0	32	0	7	0	10	0	17
Escherichia coli K12	2	0	0	0	271	1	33	0	321	4	85	0	294	5	121
Escherichia coli O157	2	0	0	0	272	0	32	0	326	1	85	0	289	2	130
Escherichia coli O157_EDL933	2	0	0	0	269	1	32	0	324	3	85	0	293	2	131
Fusobacterium nucleatum subsp. Nucleatum ATCC25586	0	0	0	0	100	1	20	0	152	0	40	0	91	0	81
Haemophilus influenzae Rd-KW20	1	0	0	0	90	0	20	0	135	0	48	0	96	0	65
Halobacterium sp. NRC1	1	0	2	0	113	0	9	2	131	3	53	2	36	0	77
Helicobacter pylori 26695	0	0	0	0	68	0	19	1	87	0	34	0	36	0	65
Helicobacter pylori J99	0	0	0	0	67	0	17	3	87	0	34	0	36	0	66
Lactococcus lactis	0	0	0	0	59	4	17	0	154	1	70	1	132	3	55
Listeria innocua Clip11262	0	0	0	0	98	0	29	0	200	2	67	0	223	0	90
Mesorhizobium loti	0	0	5	0	234	7	24	0	450	11	81	2	240	2	157
Methanobacterium thermoautotrophicum deltaH	1	0	4	0	151	0	10	0	98	0	49	0	39	0	106
Methanococcus jannaschii DSM2661	1	0	5	0	133	0	17	0	97	4	45	0	42	2	94
Methanopyrus kandleri AV19	1	0	4	0	110	0	17	0	102	0	43	0	32	0	89
Methanosarcina acetivorans str. C2A	1	0	2	0	228	10	19	1	196	9	60	2	89	2	132
Mycobacterium leprae	1	0	0	0	65	0	21	0	121	0	49	0	64	0	69
Mycobacterium tuberculosis CDC1551	1	0	0	0	165	1	38	1	181	2	63	0	111	0	121
Mycobacterium tuberculosis H37Rv	1	0	0	0	173	0	38	2	191	0	65	0	111	0	117
Mycoplasma genitalium G37	0	0	0	0	20	0	5	0	15	0	21	0	26	0	13
Mycoplasma pneumoniae M129	0	0	0	0	19	1	5	0	24	0	21	0	37	0	13
Mycoplasma pulmonis	0	0	0	0	29	0	5	0	25	0	24	0	62	0	12
Neisseria meningitidis serogroup A strain Z2491	1	0	1	0	105	0	25	0	136	0	44	0	57	0	74
Neisseria meningitidis serogroup B strain MC58	1	0	1	0	106	1	22	3	136	0	43	0	56	0	73
Nostoc sp. PCC 7120	1	0	2	0	172	8	28	0	212	0	63	0	134	1	136
Pasteurella multocida Pm70	1	0	0	0	112	1	24	0	155	1	57	0	159	0	89
Pseudomonas aeruginosa PAO1	2	0	3	0	275	0	31	1	414	1	98	1	144	0	152
Pyrobaculum aerophilum	1	0	2	0	148	0	7	0	142	0	49	0	71	0	82
Pyrococcus abyssi GE5	1	0	3	0	117	2	17	0	127	0	47	0	76	0	59
Pyrococcus horikoshii OT3	1	0	3	0	99	0	17	0	104	0	45	0	79	1	56
Ralstonia solanacearum	2	0	2	0	174	2	23	0	242	0	72	0	115	2	107
Rickettsia conorii Malish 7	0	0	0	0	77	0	18	0	32	0	22	0	20	0	31
Rickettsia prowazekii MadridE	0	0	0	0	72	0	14	0	25	0	17	0	19	0	25
Salmonella typhimurium LT2	2	0	0	0	255	3	33	0	326	1	80	1	293	10	147
Sinorhizobium meliloti 1021	0	0	1	0	133	0	24	0	224	4	71	0	94	0	105
Staphylococcus aureus subsp. Aureus N315	0	0	1	0	102	1	23	0	209	3	66	0	122	0	78
Streptococcus pneumoniae TIGR4	0	0	0	0	46	0	20	0	135	1	60	0	176	2	44
Streptococcus pyogenes	0	0	0	0	56	1	15	1	94	2	59	0	119	3	34
Sulfolobus solfataricus	1	0	2	1	164	7	9	0	186	3	55	5	90	6	81
Synechocystis sp PCC6803	0	0	1	0	134	1	23	1	159	5	58	1	96	9	106
Thermoplasma acidophilum	0	0	0	0	109	0	7	0	113	0	46	0	70	0	70
Thermoplasma volcanium	0	0	0	0	109	3	7	1	102	3	44	0	60	3	66
Thermotoga maritima MSB8	0	0	0	0	110	0	18	0	150	0	50	0	155	0	48
Treponema pallidum Nichols	0	0	0	0	35	0	13	0	24	0	22	0	38	3	19
Ureaplasma urealyticum serovar3	0	0	0	0	16	0	4	0	20	0	21	0	15	0	9
Vibrio cholerae chromosome I	1	0	1	0	129	2	30	0	184	1	59	1	101	6	113
Vibrio cholerae chromosome II	0	0	0	0	37	0	3	2	55	1	10	2	52	0	14
Xylella fastidiosa 9a5c	1	0	0	0	81	5	26	1	113	3	40	0	70	6	72
Yersinia pestis CO92	1	0	0	0	173	1	31	0	276	1	68	1	232	4	108

Table S7: Gene count in each of the COG categories (excluding the COG-None Categories)

DIMOB Islands (all ORFs included)

ORG	H_ISLAND	I_OUTSIDE	I_ISLAND	J_OUTSIDE	J_ISLAND	K_OUTSIDE	K_ISLAND	L_OUTSIDE	L_ISLAND	M_OUTSIDE	M_ISLAND	N_OUTSIDE	N_ISLAND	O_OUTSIDE	O_ISLAND
Aeropyrum pernix K1	0	37	0	156	0	70	0	62	0	23	0	11	0	55	0
Agrobacterium tumefaciens C58 Cereon Circular	0	79	0	137	0	144	3	87	2	140	2	44	0	101	1
Agrobacterium tumefaciens C58 Cereon Linear	0	35	1	18	0	51	0	28	11	56	5	3	0	20	0
Aquifex aeolicus VF5	0	44	0	135	1	42	1	78	1	107	0	44	0	70	1
Archaeoglobus fulgidus DSM4304	0	76	2	157	0	100	0	93	3	47	0	19	0	67	0
Bacillus halodurans C125	0	96	1	112	42	208	12	165	19	124	4	53	0	92	2
Bacillus subtilis 168	0	94	0	154	2	207	2	111	20	168	5	55	0	93	2
Borrelia burgdorferi B31	0	16	0	119	0	20	0	47	0	52	0	47	0	30	0
Brucella melitensis choromosome I	0	74	0	130	2	91	3	86	15	109	8	3	0	96	4
Brucella melitensis choromosome II	0	21	0	25	0	51	2	16	5	46	0	30	0	21	0
Buchnera sp. APS	0	14	0	112	0	17	0	38	0	26	0	22	0	34	0
Campylobacter jejuni NCTC11168	0	37	0	125	0	37	0	62	0	119	0	50	0	70	0
Caulobacter crescentus	0	120	0	157	3	174	1	124	7	187	1	62	0	119	2
Chlamydia trachomatis D	0	35	0	113	0	21	0	52	1	38	0	14	0	35	0
Chlamydophila pneumoniae CWL029	0	29	1	116	0	21	0	52	1	37	0	14	0	35	0
Clostridium acetobutylicum ATCC824	0	70	0	159	0	190	5	129	6	158	2	68	0	83	0
Corynebacterium glutamicum	0	64	2	133	0	137	3	102	7	78	11	2	0	78	4
Deinococcus radiodurans chrom 1	2	79	0	149	0	98	1	101	5	79	0	11	0	91	0
Deinococcus radiodurans chrom 2	0	11	0	2	0	9	1	4	1	16	0	4	0	13	1
Escherichia coli K12	5	89	2	167	2	189	10	161	34	217	17	80	2	126	2
Escherichia coli O157	0	95	1	168	1	199	11	178	41	234	4	86	1	135	4
Escherichia coli O157_EDL933	0	95	1	168	2	199	13	172	48	231	11	86	1	138	5
Fusobacterium nucleatum subsp. Nucleatum ATCC25586	0	51	1	136	1	78	0	111	10	103	0	6	0	51	0
Haemophilus influenzae Rd-KW20	0	41	0	140	0	65	2	92	1	108	0	6	0	76	1
Halobacterium sp. NRC1	6	55	0	138	4	86	6	79	19	44	8	32	1	66	1
Helicobacter pylori 26695	0	42	0	119	0	23	0	84	12	96	0	50	0	62	0
Helicobacter pylori J99	0	40	1	119	0	23	1	79	6	94	1	47	0	63	0
Lactococcus lactis	0	49	0	109	28	117	8	106	13	94	1	8	0	44	2
Listeria innocua Clip11262	0	54	0	145	0	178	3	113	13	96	0	42	0	60	0
Mesorhizobium loti	4	158	4	170	1	230	8	162	37	228	7	49	2	167	6
Methanobacterium thermoautotrophicum deltaH	0	25	0	140	0	68	0	71	0	51	0	3	0	61	0
Methanococcus jannaschii DSM2661	0	15	0	150	0	59	0	81	5	34	0	19	0	46	0
Methanopyrus kandleri AV19	0	17	0	161	0	49	0	64	0	26	0	3	0	42	0
Methanosarcina acetivorans str. C2A	4	35	3	158	3	121	6	178	25	81	4	42	0	116	3
Mycobacterium leprae	0	79	0	119	0	59	0	62	0	64	0	5	0	61	0
Mycobacterium tuberculosis CDC1551	0	130	1	142	0	135	0	141	15	102	0	8	0	95	0
Mycobacterium tuberculosis H37Rv	2	130	1	136	5	135	0	154	17	101	5	9	0	96	0
Mycoplasma genitalium G37	0	9	0	100	0	14	0	40	0	12	0	0	0	20	0
Mycoplasma pneumoniae M129	0	9	0	101	0	14	0	41	2	12	0	0	0	20	0
Mycoplasma pulmonis	0	8	0	101	2	21	0	55	7	8	0	0	0	17	1
Neisseria meningitidis serogroup A strain Z2491	0	43	0	147	0	64	1	140	1	129	0	27	0	68	0
Neisseria meningitidis serogroup B strain MC58	0	50	0	116	35	63	1	120	13	125	0	26	0	68	1
Nostoc sp. PCC 7120	0	68	0	158	0	102	3	145	9	188	0	28	0	158	2
Pasteurella multocida Pm70	0	42	0	152	0	83	2	96	7	141	0	8	0	90	0
Pseudomonas aeruginosa PAO1	1	159	2	179	0	204	2	129	8	211	0	126	3	173	1
Pyrobaculum aerophilum	0	43	0	145	0	84	0	76	0	34	0	6	0	70	0
Pyrococcus abyssi GE5	0	19	0	151	1	85	0	62	2	48	1	27	0	49	0
Pyrococcus horikoshii OT3	0	18	0	155	0	82	1	76	1	43	0	28	0	49	0
Ralstonia solanacearum	0	101	1	152	0	141	7	140	18	153	1	55	0	119	5
Rickettsia conorii Malish 7	0	41	0	126	0	25	0	71	0	83	0	4	0	58	0
Rickettsia prowazekii MadridE	0	28	0	124	0	20	0	57	0	73	0	2	0	52	0
Salmonella typhimurium LT2	1	83	1	168	1	198	11	138	22	229	10	81	5	145	5
Sinorhizobium meliloti 1021	0	77	2	150	3	94	2	108	7	111	2	48	0	108	2
Staphylococcus aureus subsp. Aureus N315	2	66	0	144	5	126	3	121	20	102	3	4	1	69	2
Streptococcus pneumoniae TIGR4	0	33	0	141	2	114	2	111	6	87	0	5	0	54	0
Streptococcus pyogenes	0	48	1	138	2	102	2	104	5	64	2	10	0	50	0
Sulfolobus solfataricus	0	70	3	153	1	100	2	124	20	56	6	14	0	72	2
Synechocystis sp PCC6803	3	52	0	140	3	62	6	98	18	132	8	26	4	121	3
Thermoplasma acidophilum	0	51	0	132	0	78	0	68	1	34	5	13	0	46	0
Thermoplasma volcanium	1	46	2	129	3	70	2	70	39	32	8	13	0	46	3
Thermotoga maritima MSB8	0	30	0	133	0	74	0	87	0	64	0	57	0	51	0
Treponema pallidum Nichols	1	18	0	117	1	27	0	58	1	63	1	44	0	42	1
Ureaplasma urealyticum serovar3	0	8	0	103	0	19	0	50	1	6	0	0	0	19	0
Vibrio cholerae chromosome I	0	60	3	156	0	118	7	111	11	130	7	83	7	100	2
Vibrio cholerae chromosome II	0	13	1	10	6	33	3	17	15	23	2	6	0	23	3
Xylella fastidiosa 9a5c	2	49	3	134	0	76	7	116	6	137	1	52	0	91	0
Yersinia pestis CO92	6	74	1	117	48	151	14	133	14	189	5	106	6	117	3

Table S7: Gene count in each of the COG categories (excluding the COG-None Categories)

DIMOB Islands (all ORFs included)

ORG	P_OUTSIDE	P_ISLAND	Q_OUTSIDE	Q_ISLAND	R_OUTSIDE	R_ISLAND	S_OUTSIDE	S_ISLAND	T_OUTSIDE	T_ISLAND	U_OUTSIDE	U_ISLAND	V_OUTSIDE	V_ISLAND
Aeropyrum pernix K1	46	0	10	0	203	0	117	0	12	0	11	0	18	0
Agrobacterium tumefaciens C58 Cereon Circular	124	2	29	0	251	5	260	4	77	0	28	0	25	0
Agrobacterium tumefaciens C58 Cereon Linear	74	0	26	0	135	7	125	2	26	0	5	1	12	0
Aquifex aeolicus VF5	54	2	18	0	155	1	95	0	38	0	21	0	9	0
Archaeoglobus fulgidus DSM4304	80	0	17	0	284	4	245	3	45	0	15	0	20	0
Bacillus halodurans C125	151	3	37	1	323	8	330	22	107	1	19	2	53	0
Bacillus subtilis 168	159	0	51	0	330	7	326	15	96	0	25	0	48	3
Borrelia burgdorferi B31	17	0	0	0	70	0	43	0	13	0	12	0	9	0
Brucella melitensis choromosome I	64	1	18	0	180	2	194	5	49	0	22	0	21	0
Brucella melitensis choromosome II	56	1	17	0	81	2	60	4	18	1	8	0	15	4
Buchnera sp. APS	15	0	1	0	27	0	15	0	5	0	11	0	4	0
Campylobacter jejuni NCTC11168	81	0	19	0	133	0	93	0	31	0	26	0	26	0
Caulobacter crescentus	112	2	66	0	287	2	329	5	81	0	49	1	40	0
Chlamydia trachomatis D	18	0	2	0	57	1	31	2	10	2	19	0	3	0
Chlamydophila pneumoniae CWL029	22	0	2	1	58	0	40	0	12	0	19	0	3	0
Clostridium acetobutylicum ATCC824	97	1	20	0	310	3	269	5	99	0	16	0	60	0
Corynebacterium glutamicum	143	6	31	1	217	9	169	7	52	3	16	0	31	1
Deinococcus radiodurans chrom 1	81	0	27	1	242	6	222	1	69	1	17	1	39	0
Deinococcus radiodurans chrom 2	18	1	9	0	23	1	13	0	12	0	0	0	11	0
Escherichia coli K12	175	15	45	1	329	18	309	13	96	1	37	0	47	1
Escherichia coli O157	205	5	42	1	393	20	393	20	107	5	46	1	53	2
Escherichia coli O157_EDL933	207	11	43	1	379	30	384	26	100	6	46	2	52	2
Fusobacterium nucleatum subsp. Nucleatum ATCC25586	81	0	12	0	173	3	129	0	25	0	26	0	33	0
Haemophilus influenzae Rd-KW20	77	0	13	0	133	2	135	0	32	0	26	0	16	0
Halobacterium sp. NRC1	77	2	17	1	191	5	144	10	32	0	12	0	20	0
Helicobacter pylori 26695	49	0	8	0	102	2	73	1	15	1	33	8	30	2
Helicobacter pylori J99	50	0	8	0	108	0	65	3	16	0	32	7	27	0
Lactococcus lactis	80	1	15	0	178	7	184	9	37	1	12	1	40	1
Listeria innocua Clip11262	111	0	31	0	257	2	287	3	80	0	19	0	55	3
Mesorhizobium loti	176	1	77	2	486	15	560	20	108	2	60	8	52	2
Methanobacterium thermoautotrophicum deltaH	72	0	8	0	233	0	217	0	31	0	12	0	16	0
Methanococcus jannaschii DSM2661	64	0	9	0	221	3	208	4	10	0	12	0	15	0
Methanopyrus kandleri AV19	30	0	9	0	207	0	197	0	14	0	13	0	5	0
Methanosarcina acetivorans str. C2A	158	5	21	4	377	21	341	15	55	0	21	1	56	4
Mycobacterium leprae	46	0	37	0	101	0	78	0	32	0	15	0	13	0
Mycobacterium tuberculosis CDC1551	110	1	82	1	298	3	230	6	81	0	17	1	38	0
Mycobacterium tuberculosis H37Rv	114	0	84	1	302	4	228	11	80	0	17	1	38	0
Mycoplasma genitalium G37	17	0	0	0	41	0	15	0	3	0	6	0	8	0
Mycoplasma pneumoniae M129	17	0	0	0	45	1	17	0	3	0	7	0	19	0
Mycoplasma pulmonis	16	0	0	0	52	0	27	1	3	0	9	0	29	0
Neisseria meningitidis serogroup A strain Z2491	81	1	16	0	148	1	168	0	26	0	25	0	15	1
Neisseria meningitidis serogroup B strain MC58	78	4	15	3	138	2	161	4	26	2	19	11	17	1
Nostoc sp. PCC 7120	208	2	72	3	369	6	359	5	137	1	34	0	70	4
Pasteurella multocida Pm70	115	4	16	1	181	1	171	3	44	0	38	0	26	1
Pseudomonas aeruginosa PAO1	266	0	91	0	461	5	477	2	138	2	70	0	64	0
Pyrobaculum aerophilum	59	0	12	0	242	0	151	0	22	0	11	0	22	0
Pyrococcus abyssi GE5	71	0	11	1	255	5	187	2	15	0	12	0	22	0
Pyrococcus horikoshii OT3	55	0	15	0	240	0	184	0	17	0	12	0	19	0
Ralstonia solanacearum	107	0	53	3	292	3	272	7	85	1	57	1	25	1
Rickettsia conorii Malish 7	22	0	9	0	92	0	69	0	22	0	32	0	23	0
Rickettsia prowazekii MadridE	20	0	10	0	60	0	48	0	14	0	31	0	9	0
Salmonella typhimurium LT2	174	2	52	0	341	37	334	28	91	2	43	1	45	1
Sinorhizobium meliloti 1021	109	1	28	1	252	7	318	6	33	0	37	0	22	3
Staphylococcus aureus subsp. Aureus N315	127	3	24	4	223	7	215	8	48	0	18	0	42	0
Streptococcus pneumoniae TIGR4	77	0	10	0	157	1	147	3	40	0	17	0	55	0
Streptococcus pyogenes	58	0	7	0	136	2	140	4	39	0	14	0	37	2
Sulfolobus solfataricus	58	1	30	1	269	29	149	16	13	1	11	0	21	0
Synechocystis sp PCC6803	146	5	35	1	250	14	205	8	102	8	26	0	38	1
Thermoplasma acidophilum	36	0	15	1	154	1	112	0	9	0	12	0	14	0
Thermoplasma volcanium	32	1	11	2	139	9	106	5	9	1	11	0	11	6
Thermotoga maritima MSB8	69	0	13	0	193	0	131	0	47	0	12	0	31	0
Treponema pallidum Nichols	23	0	2	0	79	1	49	2	22	0	12	0	6	1
Ureaplasma urealyticum serovar3	25	0	0	0	45	0	28	0	3	0	7	0	16	0
Vibrio cholerae chromosome I	101	0	31	3	198	6	197	4	92	2	34	0	37	0
Vibrio cholerae chromosome II	52	1	7	0	80	11	70	10	22	0	5	0	8	2
Xylella fastidiosa 9a5c	64	0	21	0	188	10	175	4	47	0	46	0	30	0
Yersinia pestis CO92	182	7	44	0	246	13	323	32	78	2	43	14	40	2

Table S7: Gene count in each of the COG categories (excluding the COG-None Categories)

DINUC Islands (ORFs < 300bps excluded)

ORG	A_OUTSIDE	A_ISLAND	B_OUTSIDE	B_ISLAND	C_OUTSIDE	C_ISLAND	D_OUTSIDE	D_ISLAND	E_OUTSIDE	E_ISLAND	F_OUTSIDE	F_ISLAND	G_OUTSIDE	G_ISLAND	H_OUTSIDE
Aeropyrum pernix K1	1	0	2	0	99	3	3	1	117	19	33	3	55	3	53
Agrobacterium tumefaciens C58 Cereon Circular	0	0	1	0	105	18	16	0	209	15	64	5	107	6	74
Agrobacterium tumefaciens C58 Cereon Linear	0	0	0	0	53	5	9	0	152	7	16	2	97	4	40
Aquifex aeolicus VF5	1	0	2	0	72	40	16	0	94	15	41	3	38	4	77
Archaeoglobus fulgidus DSM4304	1	0	3	1	172	22	13	1	122	21	43	4	39	9	83
Bacillus halodurans C125	0	0	0	1	145	9	31	2	256	12	65	6	214	13	103
Bacillus subtilis 168	0	0	1	0	156	5	32	1	271	5	72	6	211	15	101
Borrelia burgdorferi B31	0	0	0	0	19	4	11	3	21	5	16	4	35	5	10
Brucella melitensis choromosome I	0	0	1	0	89	9	18	1	158	5	44	3	68	5	82
Brucella melitensis choromosome II	0	0	0	0	52	10	6	0	129	13	18	0	68	13	26
Buchnera sp. APS	1	0	0	0	38	1	8	0	53	1	20	5	30	0	29
Campylobacter jejuni NCTC11168	0	0	0	0	80	12	14	2	127	5	41	2	32	7	66
Caulobacter crescentus	0	0	2	0	136	17	20	2	196	16	55	9	121	9	78
Chlamydia trachomatis D	0	0	1	0	36	3	8	1	42	9	15	1	31	4	33
Chlamydophila pneumoniae CWL029	0	0	1	0	39	0	8	1	47	8	20	1	32	3	40
Clostridium acetobutylicum ATCC824	0	0	0	0	93	14	30	4	206	9	66	4	181	22	83
Corynebacterium glutamicum	1	0	0	0	92	11	14	3	173	4	58	3	114	6	91
Deinococcus radiodurans chrom 1	0	0	1	0	89	22	16	2	174	21	60	5	80	4	57
Deinococcus radiodurans chrom 2	0	0	0	0	19	1	1	1	32	0	7	0	9	1	16
Escherichia coli K12	2	0	0	0	243	20	29	1	311	13	77	8	287	8	111
Escherichia coli O157	2	0	0	0	252	9	30	0	319	7	79	6	278	5	123
Escherichia coli O157_EDL933	2	0	0	0	251	9	30	0	318	8	80	5	279	7	123
Fusobacterium nucleatum subsp. Nucleatum ATCC25586	0	0	0	0	87	6	19	1	127	23	35	5	75	14	74
Haemophilus influenzae Rd-KW20	1	0	0	0	78	7	16	3	127	8	44	4	77	18	62
Halobacterium sp. NRC1	1	0	2	0	99	9	9	2	123	8	48	6	35	1	70
Helicobacter pylori 26695	0	0	0	0	66	0	18	1	84	2	34	0	35	1	64
Helicobacter pylori J99	0	0	0	0	64	1	16	3	82	5	33	1	36	0	64
Lactococcus lactis	0	0	0	0	55	4	14	3	152	2	64	5	127	7	52
Listeria innocua Clip11262	0	0	0	0	84	10	24	5	186	16	63	3	199	17	82
Mesorhizobium loti	0	0	5	0	217	20	22	1	417	36	75	6	218	20	135
Methanobacterium thermoautotrophicum deltaH	1	0	1	0	123	20	10	0	93	3	46	2	33	5	82
Methanococcus jannaschii DSM2661	1	0	1	0	97	24	16	1	91	8	40	4	42	2	79
Methanopyrus kandleri AV19	1	0	2	0	88	19	15	2	90	10	40	2	26	6	71
Methanosarcina acetivorans str. C2A	1	0	1	0	188	35	18	2	179	23	52	9	85	6	108
Mycobacterium leprae	1	0	0	0	56	8	18	3	115	5	45	3	61	3	62
Mycobacterium tuberculosis CDC1551	1	0	0	0	137	22	31	3	172	10	60	2	100	10	109
Mycobacterium tuberculosis H37Rv	1	0	0	0	148	18	31	3	184	6	60	4	101	10	103
Mycoplasma genitalium G37	0	0	0	0	14	6	5	0	14	1	18	3	21	4	8
Mycoplasma pneumoniae M129	0	0	0	0	19	1	5	0	19	5	21	0	30	5	12
Mycoplasma pulmonis	0	0	0	0	28	0	4	0	24	1	23	1	48	12	12
Neisseria meningitidis serogroup A strain Z2491	1	0	1	0	92	8	21	2	131	3	41	3	53	2	73
Neisseria meningitidis serogroup B strain MC58	1	0	1	0	99	2	20	2	135	1	40	3	53	2	72
Nostoc sp. PCC 7120	1	0	1	1	145	26	21	3	179	33	55	7	117	18	116
Pasteurella multocida Pm70	1	0	0	0	104	3	21	2	153	3	55	2	149	9	83
Pseudomonas aeruginosa PAO1	2	0	3	0	233	33	25	4	385	30	89	10	133	10	132
Pyrobaculum aerophilum	1	0	1	1	122	14	7	0	133	8	44	4	67	3	74
Pyrococcus abyssi GE5	1	0	1	0	97	18	14	3	109	18	43	3	72	4	54
Pyrococcus horikoshii OT3	1	0	1	0	82	16	16	1	95	9	41	3	78	2	50
Ralstonia solanacearum	2	0	2	0	163	7	21	1	209	28	70	2	98	18	95
Rickettsia conorii Malish 7	0	0	0	0	62	11	13	2	30	0	18	1	18	2	24
Rickettsia prowazekii MadridE	0	0	0	0	55	16	14	0	25	0	15	2	16	3	24
Salmonella typhimurium LT2	2	0	0	0	230	16	30	0	315	10	80	1	277	19	139
Sinorhizobium meliloti 1021	0	0	1	0	117	15	20	0	210	16	68	2	88	5	93
Staphylococcus aureus subsp. Aureus N315	0	0	1	0	97	2	20	1	204	7	62	3	114	4	75
Streptococcus pneumoniae TIGR4	0	0	0	0	42	1	19	0	134	1	54	6	168	5	43
Streptococcus pyogenes	0	0	0	0	53	1	14	2	86	9	56	3	111	8	34
Sulfolobus solfataricus	1	0	2	1	144	20	9	0	177	10	51	7	79	15	80
Synechocystis sp PCC6803	0	0	1	0	123	7	19	0	154	8	56	2	94	9	102
Thermoplasma acidophilum	0	0	0	0	92	9	6	1	78	35	44	1	66	3	55
Thermoplasma volcanium	0	0	0	0	92	13	7	1	99	6	43	0	54	9	59
Thermotoga maritima MSB8	0	0	0	0	88	13	16	1	124	26	46	3	122	33	44
Treponema pallidum Nichols	0	0	0	0	32	1	11	2	22	2	21	1	36	4	18
Ureaplasma urealyticum serovar3	0	0	0	0	16	0	4	0	20	0	20	1	14	0	8
Vibrio cholerae chromosome I	1	0	1	0	108	15	28	0	180	5	55	5	92	13	104
Vibrio cholerae chromosome II	0	0	0	0	37	0	1	0	49	7	10	2	52	0	13
Xylella fastidiosa 9a5c	1	0	0	0	72	12	23	2	109	7	39	1	68	7	66
Yersinia pestis CO92	1	0	0	0	158	11	29	1	272	5	66	3	208	25	100

Table S7: Gene count in each of the COG categories (excluding the COG-None Categories)

DINUC Islands (ORFs < 300bps excluded)

ORG	H_ISLAND	I_OUTSIDE	I_ISLAND	J_OUTSIDE	J_ISLAND	K_OUTSIDE	K_ISLAND	L_OUTSIDE	L_ISLAND	M_OUTSIDE	M_ISLAND	N_OUTSIDE	N_ISLAND	O_OUTSIDE	O_ISLAND
Aeropyrum pernix K1	3	33	4	113	22	52	4	49	13	13	10	11	0	42	13
Agrobacterium tumefaciens C58 Cereon Circular	9	72	5	93	34	122	17	79	7	132	10	15	28	88	12
Agrobacterium tumefaciens C58 Cereon Linear	1	32	4	13	2	43	3	22	13	55	6	3	0	17	2
Aquifex aeolicus VF5	3	40	3	102	17	34	6	67	11	88	19	32	10	60	11
Archaeoglobus fulgidus DSM4304	3	69	7	121	8	66	7	84	8	35	11	17	2	50	8
Bacillus halodurans C125	3	87	6	89	40	182	21	145	28	109	17	33	18	89	3
Bacillus subtilis 168	3	85	5	95	32	168	23	104	23	162	10	47	6	86	6
Borrelia burgdorferi B31	2	14	1	65	35	16	3	41	6	47	4	34	11	24	5
Brucella melitensis choromosome I	2	67	5	81	38	76	8	80	16	106	11	1	1	92	5
Brucella melitensis choromosome II	1	21	0	19	2	45	7	15	4	38	8	13	16	18	1
Buchnera sp. APS	3	12	1	65	28	12	1	31	5	23	3	11	9	30	2
Campylobacter jejuni NCTC11168	5	31	2	86	17	28	6	57	2	98	21	41	7	64	1
Caulobacter crescentus	13	105	13	109	32	141	19	101	20	167	21	29	30	97	19
Chlamydia trachomatis D	3	31	3	77	21	16	4	45	8	32	5	14	0	31	4
Chlamydophila pneumoniae CWL029	0	25	4	73	29	20	1	48	5	31	6	14	0	33	2
Clostridium acetobutylicum ATCC824	20	59	6	101	33	150	26	116	15	146	8	42	24	75	5
Corynebacterium glutamicum	4	57	7	93	24	120	13	89	14	72	17	2	0	69	10
Deinococcus radiodurans chrom 1	9	75	3	118	12	89	6	90	14	69	10	8	3	83	6
Deinococcus radiodurans chrom 2	0	11	0	2	0	10	0	4	1	7	9	4	0	13	0
Escherichia coli K12	13	87	3	109	35	158	22	142	40	190	39	66	14	109	9
Escherichia coli O157	6	88	4	107	36	166	24	151	50	209	27	68	18	119	10
Escherichia coli O157_EDL933	6	91	1	108	35	165	23	154	49	211	26	71	13	123	10
Fusobacterium nucleatum subsp. Nucleatum ATCC25586	6	42	9	85	30	62	14	98	12	80	21	4	2	42	7
Haemophilus influenzae Rd-KW20	1	34	6	95	27	54	7	82	4	101	6	6	0	68	4
Halobacterium sp. NRC1	12	54	1	92	28	66	6	72	24	44	7	25	8	54	8
Helicobacter pylori 26695	0	40	1	96	1	21	0	79	14	92	3	46	3	55	4
Helicobacter pylori J99	0	40	0	97	0	21	1	73	9	91	4	43	2	56	4
Lactococcus lactis	3	39	8	82	31	103	16	94	18	76	19	7	0	41	3
Listeria innocua Clip11262	7	46	6	89	29	150	19	102	17	84	11	28	10	56	1
Mesorhizobium loti	22	141	16	121	31	202	22	138	46	211	22	38	11	148	19
Methanobacterium thermoautotrophicum deltaH	21	24	0	91	24	47	10	55	15	43	6	2	1	50	5
Methanococcus jannaschii DSM2661	13	15	0	86	36	37	10	74	11	24	10	10	9	38	4
Methanopyrus kandleri AV19	14	15	2	120	18	34	6	52	12	22	4	3	0	34	6
Methanosarcina acetivorans str. C2A	19	32	4	123	9	91	19	162	34	76	9	39	0	104	9
Mycobacterium leprae	5	68	10	76	28	48	8	55	5	58	6	4	1	52	8
Mycobacterium tuberculosis CDC1551	8	113	13	101	21	116	9	130	21	91	11	5	3	78	14
Mycobacterium tuberculosis H37Rv	12	118	11	100	21	117	9	150	15	90	16	5	4	78	16
Mycoplasma genitalium G37	5	7	1	58	26	13	1	33	7	11	1	0	0	18	2
Mycoplasma pneumoniae M129	1	7	1	70	15	14	0	39	4	11	1	0	0	20	0
Mycoplasma pulmonis	0	5	2	62	25	17	4	49	11	8	0	0	0	15	3
Neisseria meningitidis serogroup A strain Z2491	0	42	0	92	33	55	4	122	9	118	10	23	4	62	1
Neisseria meningitidis serogroup B strain MC58	0	47	0	99	30	55	2	110	14	117	8	22	4	62	3
Nostoc sp. PCC 7120	14	59	7	98	36	84	17	129	20	165	23	22	4	132	24
Pasteurella multocida Pm70	4	40	1	98	34	69	10	86	13	127	14	7	1	82	4
Pseudomonas aeruginosa PAO1	18	145	12	111	46	169	24	114	16	185	26	109	18	131	33
Pyrobaculum aerophilum	4	38	5	119	5	56	3	59	13	27	7	5	1	59	6
Pyrococcus abyssi GE5	2	16	3	119	10	61	11	56	8	40	9	15	12	44	4
Pyrococcus horikoshii OT3	4	18	0	128	3	67	5	73	2	33	10	12	16	43	5
Ralstonia solanacearum	9	91	9	97	35	110	25	126	23	140	12	40	14	110	9
Rickettsia conorii Malish 7	5	30	3	83	25	18	5	59	6	73	5	4	0	47	8
Rickettsia prowazekii MadridE	1	26	1	76	30	13	4	49	5	68	5	2	0	44	6
Salmonella typhimurium LT2	6	79	3	108	36	162	28	128	20	199	37	74	10	113	28
Sinorhizobium meliloti 1021	11	72	4	104	28	79	8	98	13	106	7	31	15	100	6
Staphylococcus aureus subsp. Aureus N315	4	64	0	90	32	110	10	109	19	100	5	4	1	64	2
Streptococcus pneumoniae TIGR4	1	30	0	96	22	96	9	101	9	80	7	4	0	45	7
Streptococcus pyogenes	0	45	1	88	28	81	14	94	10	62	4	7	2	48	0
Sulfolobus solfataricus	0	67	6	109	19	79	4	113	29	52	10	12	2	63	4
Synechocystis sp PCC6803	4	50	1	101	23	61	5	95	20	125	14	25	4	115	5
Thermoplasma acidophilum	10	49	2	85	25	59	10	54	11	27	10	13	0	40	4
Thermoplasma volcanium	4	41	7	88	22	54	8	57	36	32	8	10	3	37	8
Thermotoga maritima MSB8	4	25	3	98	16	60	9	76	7	50	14	49	4	46	1
Treponema pallidum Nichols	2	16	1	74	25	22	4	54	5	59	5	31	11	40	1
Ureaplasma urealyticum serovar3	1	7	0	69	17	17	1	48	3	6	0	0	0	18	1
Vibrio cholerae chromosome I	7	59	3	92	41	96	22	98	19	123	13	78	11	87	7
Vibrio cholerae chromosome II	1	12	2	8	5	29	3	15	15	21	4	6	0	21	3
Xylella fastidiosa 9a5c	7	46	4	96	21	60	14	104	10	129	7	50	0	78	12
Yersinia pestis CO92	13	72	1	102	39	127	21	123	10	173	19	81	27	106	7

Table S7: Gene count in each of the COG categories (excluding the COG-None Categories)

DINUC Islands (ORFs < 300bps excluded)

ORG	P_OUTSIDE	P_ISLAND	Q_OUTSIDE	Q_ISLAND	R_OUTSIDE	R_ISLAND	S_OUTSIDE	S_ISLAND	T_OUTSIDE	T_ISLAND	U_OUTSIDE	U_ISLAND	V_OUTSIDE	V_ISLAND
Aeropyrum pernix K1	40	6	7	3	177	24	92	16	10	1	5	2	17	1
Agrobacterium tumefaciens C58 Cereon Circular	117	7	27	2	233	19	219	29	68	8	24	1	25	0
Agrobacterium tumefaciens C58 Cereon Linear	73	0	24	2	125	16	104	13	23	2	5	1	12	0
Aquifex aeolicus VF5	48	7	14	4	132	22	84	9	36	2	17	1	8	1
Archaeoglobus fulgidus DSM4304	72	4	14	3	242	30	182	27	41	3	11	2	18	2
Bacillus halodurans C125	143	8	33	4	301	25	275	37	92	14	13	3	50	2
Bacillus subtilis 168	150	6	47	4	304	27	255	40	90	3	14	4	47	4
Borrelia burgdorferi B31	16	1	0	0	58	11	33	8	12	0	9	2	9	0
Brucella melitensis choromosome I	59	3	18	0	170	5	166	10	46	2	19	1	21	0
Brucella melitensis choromosome II	54	2	17	0	79	3	49	8	17	1	2	6	15	4
Buchnera sp. APS	13	2	0	1	23	3	12	2	4	0	10	1	4	0
Campylobacter jejuni NCTC11168	72	5	15	3	117	12	74	9	29	1	18	4	25	1
Caulobacter crescentus	89	20	55	11	246	32	258	48	69	9	25	20	38	2
Chlamydia trachomatis D	15	3	1	1	48	9	21	9	10	2	15	2	3	0
Chlamydophila pneumoniae CWL029	21	1	1	2	51	6	35	1	10	2	16	1	3	0
Clostridium acetobutylicum ATCC824	85	8	17	3	275	33	213	32	94	3	9	4	59	1
Corynebacterium glutamicum	129	10	29	2	206	17	150	14	45	9	13	1	30	2
Deinococcus radiodurans chrom 1	70	7	23	5	220	24	190	22	60	9	15	1	33	5
Deinococcus radiodurans chrom 2	17	2	9	0	18	6	13	0	12	0	0	0	11	0
Escherichia coli K12	162	21	42	2	309	29	261	22	91	2	26	9	43	5
Escherichia coli O157	192	13	41	1	341	56	312	62	96	7	29	12	50	5
Escherichia coli O157_EDL933	195	12	41	1	338	53	312	52	94	8	30	13	49	3
Fusobacterium nucleatum subsp. Nucleatum ATCC25586	68	10	8	2	150	21	98	20	19	6	19	4	31	1
Haemophilus influenzae Rd-KW20	55	16	12	0	118	13	102	8	25	4	22	1	15	1
Halobacterium sp. NRC1	56	19	17	1	179	15	122	20	29	2	6	2	14	6
Helicobacter pylori 26695	43	4	8	0	96	6	58	3	13	2	26	12	27	2
Helicobacter pylori J99	44	3	7	1	104	2	59	2	15	0	24	12	27	0
Lactococcus lactis	78	0	14	1	163	19	151	19	33	4	9	2	36	5
Listeria innocua Clip11262	102	5	26	0	241	14	222	29	74	5	13	3	55	3
Mesorhizobium loti	163	11	69	9	437	46	455	73	98	10	55	7	49	5
Methanobacterium thermoautotrophicum deltaH	55	10	6	2	203	20	180	16	23	7	9	1	15	1
Methanococcus jannaschii DSM2661	58	2	8	1	187	21	168	19	8	1	9	1	12	3
Methanopyrus kandleri AV19	25	4	8	1	169	31	149	31	12	1	7	3	2	3
Methanosarcina acetivorans str. C2A	141	15	21	4	328	57	280	44	50	3	16	5	48	11
Mycobacterium leprae	46	0	33	4	90	10	69	3	29	3	9	4	13	0
Mycobacterium tuberculosis CDC1551	104	6	76	7	263	31	191	25	77	4	15	1	32	5
Mycobacterium tuberculosis H37Rv	111	3	79	6	272	29	195	26	71	9	15	2	31	6
Mycoplasma genitalium G37	15	2	0	0	34	7	14	0	3	0	5	1	7	1
Mycoplasma pneumoniae M129	17	0	0	0	44	2	13	3	3	0	6	0	12	7
Mycoplasma pulmonis	16	0	0	0	47	5	22	3	3	0	7	1	29	0
Neisseria meningitidis serogroup A strain Z2491	73	6	12	3	141	4	143	5	20	3	17	5	14	2
Neisseria meningitidis serogroup B strain MC58	73	6	13	4	131	4	138	4	22	2	16	11	16	1
Nostoc sp. PCC 7120	168	38	58	15	316	50	270	50	122	12	23	7	52	22
Pasteurella multocida Pm70	108	8	13	4	170	8	143	12	40	3	20	14	25	2
Pseudomonas aeruginosa PAO1	236	25	76	14	411	46	380	55	126	11	49	16	49	15
Pyrobaculum aerophilum	57	0	9	3	216	23	128	12	18	3	6	1	19	3
Pyrococcus abyssi GE5	51	15	9	3	218	38	156	24	14	1	10	1	17	5
Pyrococcus horikoshii OT3	40	12	13	2	216	21	164	12	16	1	9	2	19	0
Ralstonia solanacearum	95	7	43	13	258	28	212	43	77	6	40	13	21	5
Rickettsia conorii Malish 7	16	4	8	0	69	7	46	8	15	2	29	0	19	1
Rickettsia prowazekii MadridE	14	5	10	0	54	5	40	1	12	0	27	2	8	1
Salmonella typhimurium LT2	162	6	47	1	318	47	274	37	82	8	33	5	43	3
Sinorhizobium meliloti 1021	96	11	27	2	221	29	261	19	28	2	31	2	20	5
Staphylococcus aureus subsp. Aureus N315	118	9	24	4	208	19	169	13	44	3	11	3	40	2
Streptococcus pneumoniae TIGR4	72	5	10	0	147	6	117	6	39	0	12	2	49	6
Streptococcus pyogenes	54	3	7	0	129	7	106	14	37	2	10	2	33	6
Sulfolobus solfataricus	52	6	30	1	259	34	140	21	13	1	8	2	19	2
Synechocystis sp PCC6803	133	14	35	1	243	18	179	8	93	15	22	1	36	3
Thermoplasma acidophilum	32	1	13	3	135	19	91	10	9	0	8	2	12	2
Thermoplasma volcanium	28	4	11	2	126	20	89	8	9	1	8	1	11	5
Thermotoga maritima MSB8	59	5	12	1	165	23	112	6	44	2	10	1	23	8
Treponema pallidum Nichols	21	2	2	0	63	16	38	9	20	1	11	0	6	1
Ureaplasma urealyticum serovar3	24	0	0	0	45	0	25	2	3	0	5	1	12	3
Vibrio cholerae chromosome I	92	5	28	6	187	13	162	12	88	5	29	3	34	3
Vibrio cholerae chromosome II	47	4	7	0	75	9	62	8	22	0	4	0	6	4
Xylella fastidiosa 9a5c	57	4	20	0	168	17	137	16	41	3	37	6	25	5
Yersinia pestis CO92	167	17	37	7	239	16	281	36	74	5	36	18	38	4

Table S7: Gene count in each of the COG categories (excluding the COG-None Categories)

DIMOB Islands (ORFs < 300bps excluded)

ORG	A_OUTSIDE	A_ISLAND	B_OUTSIDE	B_ISLAND	C_OUTSIDE	C_ISLAND	D_OUTSIDE	D_ISLAND	E_OUTSIDE	E_ISLAND	F_OUTSIDE	F_ISLAND	G_OUTSIDE	G_ISLAND	H_OUTSIDE
Aeropyrum pernix K1	1	0	2	0	102	0	4	0	136	0	36	0	58	0	56
Agrobacterium tumefaciens C58 Cereon Circular	0	0	1	0	123	0	16	0	222	2	67	2	111	2	83
Agrobacterium tumefaciens C58 Cereon Linear	0	0	0	0	57	1	9	0	159	0	18	0	101	0	41
Aquifex aeolicus VF5	1	0	2	0	109	3	16	0	103	6	44	0	42	0	79
Archaeoglobus fulgidus DSM4304	1	0	3	1	193	1	14	0	142	1	47	0	48	0	86
Bacillus halodurans C125	0	0	0	1	150	4	32	1	267	1	70	1	224	3	106
Bacillus subtilis 168	0	0	1	0	161	0	33	0	274	2	74	4	224	2	104
Borrelia burgdorferi B31	0	0	0	0	23	0	14	0	26	0	20	0	40	0	12
Brucella melitensis choromosome I	0	0	1	0	95	3	18	1	158	5	46	1	69	4	84
Brucella melitensis choromosome II	0	0	0	0	62	0	6	0	142	0	18	0	80	1	27
Buchnera sp. APS	1	0	0	0	39	0	8	0	54	0	25	0	30	0	32
Campylobacter jejuni NCTC11168	0	0	0	0	91	1	16	0	132	0	43	0	39	0	71
Caulobacter crescentus	0	0	2	0	152	1	22	0	209	3	61	3	129	1	91
Chlamydia trachomatis D	0	0	1	0	39	0	9	0	51	0	16	0	35	0	36
Chlamydophila pneumoniae CWL029	0	0	1	0	39	0	9	0	55	0	21	0	35	0	40
Clostridium acetobutylicum ATCC824	0	0	0	0	106	1	34	0	215	0	70	0	202	1	102
Corynebacterium glutamicum	1	0	0	0	100	3	16	1	177	0	61	0	119	1	95
Deinococcus radiodurans chrom 1	0	0	1	0	107	4	18	0	194	1	65	0	83	1	64
Deinococcus radiodurans chrom 2	0	0	0	0	20	0	2	0	32	0	7	0	10	0	16
Escherichia coli K12	2	0	0	0	262	1	30	0	317	7	85	0	289	6	118
Escherichia coli O157	2	0	0	0	261	0	30	0	324	2	85	0	279	4	129
Escherichia coli O157_EDL933	2	0	0	0	259	1	30	0	322	4	85	0	281	5	128
Fusobacterium nucleatum subsp. Nucleatum ATCC25586	0	0	0	0	93	0	20	0	147	3	40	0	89	0	80
Haemophilus influenzae Rd-KW20	1	0	0	0	85	0	19	0	135	0	48	0	95	0	63
Halobacterium sp. NRC1	1	0	2	0	107	1	9	2	129	2	52	2	36	0	76
Helicobacter pylori 26695	0	0	0	0	66	0	18	1	86	0	34	0	36	0	64
Helicobacter pylori J99	0	0	0	0	65	0	16	3	87	0	34	0	36	0	64
Lactococcus lactis	0	0	0	0	57	2	16	1	153	1	68	1	131	3	55
Listeria innocua Clip11262	0	0	0	0	94	0	29	0	200	2	66	0	215	1	89
Mesorhizobium loti	0	0	5	0	230	7	23	0	443	10	79	2	234	4	153
Methanobacterium thermoautotrophicum deltaH	1	0	1	0	143	0	10	0	96	0	48	0	37	1	103
Methanococcus jannaschii DSM2661	1	0	1	0	121	0	17	0	96	3	44	0	42	2	92
Methanopyrus kandleri AV19	1	0	2	0	107	0	17	0	100	0	42	0	32	0	85
Methanosarcina acetivorans str. C2A	1	0	1	0	210	13	19	1	194	8	60	1	89	2	123
Mycobacterium leprae	1	0	0	0	64	0	21	0	120	0	47	1	64	0	67
Mycobacterium tuberculosis CDC1551	1	0	0	0	156	3	33	1	180	2	62	0	109	1	116
Mycobacterium tuberculosis H37Rv	1	0	0	0	166	0	32	2	190	0	64	0	111	0	113
Mycoplasma genitalium G37	0	0	0	0	20	0	5	0	15	0	21	0	25	0	13
Mycoplasma pneumoniae M129	0	0	0	0	19	1	5	0	24	0	21	0	35	0	13
Mycoplasma pulmonis	0	0	0	0	28	0	4	0	25	0	24	0	60	0	12
Neisseria meningitidis serogroup A strain Z2491	1	0	1	0	93	7	23	0	133	1	44	0	55	0	73
Neisseria meningitidis serogroup B strain MC58	1	0	1	0	100	1	20	2	136	0	43	0	55	0	72
Nostoc sp. PCC 7120	1	0	2	0	165	6	24	0	211	1	62	0	131	4	130
Pasteurella multocida Pm70	1	0	0	0	106	1	23	0	155	1	56	1	158	0	87
Pseudomonas aeruginosa PAO1	2	0	3	0	266	0	28	1	414	1	98	1	143	0	148
Pyrobaculum aerophilum	1	0	2	0	136	0	7	0	141	0	48	0	70	0	78
Pyrococcus abyssi GE5	1	0	1	0	113	2	17	0	127	0	46	0	76	0	56
Pyrococcus horikoshii OT3	1	0	1	0	98	0	17	0	104	0	44	0	79	1	54
Ralstonia solanacearum	2	0	2	0	166	4	22	0	234	3	72	0	114	2	104
Rickettsia conorii Malish 7	0	0	0	0	72	1	13	2	30	0	19	0	20	0	28
Rickettsia prowazekii MadridE	0	0	0	0	71	0	14	0	25	0	17	0	19	0	25
Salmonella typhimurium LT2	2	0	0	0	243	3	30	0	324	1	81	0	286	10	144
Sinorhizobium meliloti 1021	0	0	1	0	132	0	20	0	220	6	70	0	93	0	103
Staphylococcus aureus subsp. Aureus N315	0	0	1	0	98	1	20	1	208	3	65	0	114	4	76
Streptococcus pneumoniae TIGR4	0	0	0	0	43	0	19	0	135	0	60	0	169	4	44
Streptococcus pyogenes	0	0	0	0	53	1	15	1	95	0	59	0	118	1	34
Sulfolobus solfataricus	1	0	2	1	158	6	9	0	184	3	54	4	88	6	80
Synechocystis sp PCC6803	0	0	1	0	128	2	19	0	156	6	57	1	95	8	103
Thermoplasma acidophilum	0	0	0	0	101	0	7	0	113	0	45	0	69	0	65
Thermoplasma volcanium	0	0	0	0	99	6	7	1	102	3	43	0	60	3	61
Thermotoga maritima MSB8	0	0	0	0	101	0	17	0	150	0	49	0	155	0	48
Treponema pallidum Nichols	0	0	0	0	33	0	13	0	24	0	22	0	37	3	19
Ureaplasma urealyticum serovar3	0	0	0	0	16	0	4	0	20	0	21	0	14	0	9
Vibrio cholerae chromosome I	1	0	1	0	121	2	28	0	184	1	59	1	100	5	111
Vibrio cholerae chromosome II	0	0	0	0	37	0	1	0	52	4	10	2	52	0	14
Xylella fastidiosa 9a5c	1	0	0	0	79	5	23	2	113	3	40	0	69	6	70
Yersinia pestis CO92	1	0	0	0	167	2	29	1	276	1	68	1	223	10	103

Table S7: Gene count in each of the COG categories (excluding the COG-None Categories)

DIMOB Islands (ORFs < 300bps excluded)

ORG	H_ISLAND	I_OUTSIDE	I_ISLAND	J_OUTSIDE	J_ISLAND	K_OUTSIDE	K_ISLAND	L_OUTSIDE	L_ISLAND	M_OUTSIDE	M_ISLAND	N_OUTSIDE	N_ISLAND	O_OUTSIDE	O_ISLAND
Aeropyrum pernix K1	0	37	0	135	0	56	0	62	0	23	0	11	0	55	0
Agrobacterium tumefaciens C58 Cereon Circular	0	77	0	127	0	135	4	84	2	139	3	43	0	99	1
Agrobacterium tumefaciens C58 Cereon Linear	0	35	1	15	0	46	0	24	11	56	5	3	0	19	0
Aquifex aeolicus VF5	1	43	0	117	2	38	2	77	1	106	1	42	0	70	1
Archaeoglobus fulgidus DSM4304	0	74	2	129	0	73	0	88	4	46	0	19	0	58	0
Bacillus halodurans C125	0	89	4	97	32	192	11	153	20	121	5	51	0	90	2
Bacillus subtilis 168	0	89	1	126	1	189	2	108	19	167	5	53	0	90	2
Borrelia burgdorferi B31	0	15	0	100	0	19	0	47	0	51	0	45	0	29	0
Brucella melitensis choromosome I	0	70	2	116	3	82	2	82	14	107	10	1	1	93	4
Brucella melitensis choromosome II	0	21	0	21	0	50	2	15	4	42	4	29	0	19	0
Buchnera sp. APS	0	13	0	93	0	13	0	36	0	26	0	20	0	32	0
Campylobacter jejuni NCTC11168	0	33	0	103	0	34	0	58	1	118	1	48	0	65	0
Caulobacter crescentus	0	117	1	137	4	158	2	113	8	186	2	59	0	112	4
Chlamydia trachomatis D	0	34	0	97	1	20	0	52	1	37	0	14	0	35	0
Chlamydophila pneumoniae CWL029	0	27	2	100	2	21	0	51	2	37	0	14	0	35	0
Clostridium acetobutylicum ATCC824	1	65	0	134	0	173	3	124	7	153	1	66	0	80	0
Corynebacterium glutamicum	0	63	1	117	0	129	4	97	6	87	2	2	0	75	4
Deinococcus radiodurans chrom 1	2	78	0	130	0	94	1	97	7	79	0	10	1	89	0
Deinococcus radiodurans chrom 2	0	11	0	2	0	10	0	4	1	16	0	4	0	13	0
Escherichia coli K12	6	88	2	144	0	171	9	146	36	212	17	72	8	115	3
Escherichia coli O157	0	91	1	143	0	182	8	157	44	230	6	82	4	124	5
Escherichia coli O157_EDL933	1	91	1	143	0	178	10	156	47	228	9	80	4	126	7
Fusobacterium nucleatum subsp. Nucleatum ATCC25586	0	51	0	115	0	76	0	110	0	100	1	6	0	49	0
Haemophilus influenzae Rd-KW20	0	40	0	122	0	60	1	85	1	107	0	6	0	71	1
Halobacterium sp. NRC1	6	55	0	116	4	67	5	79	17	45	6	25	8	61	1
Helicobacter pylori 26695	0	40	1	97	0	21	0	80	13	94	1	48	1	59	0
Helicobacter pylori J99	0	40	0	97	0	21	1	78	4	94	1	45	0	60	0
Lactococcus lactis	0	47	0	91	22	111	8	102	10	94	1	7	0	42	2
Listeria innocua Clip11262	0	52	0	117	1	166	3	107	12	94	1	38	0	57	0
Mesorhizobium loti	4	149	8	151	1	220	4	147	37	227	6	47	2	161	6
Methanobacterium thermoautotrophicum deltaH	0	24	0	115	0	57	0	67	3	49	0	3	0	55	0
Methanococcus jannaschii DSM2661	0	15	0	121	1	47	0	80	5	34	0	19	0	42	0
Methanopyrus kandleri AV19	0	17	0	138	0	40	0	64	0	26	0	3	0	40	0
Methanosarcina acetivorans str. C2A	4	34	2	129	3	102	8	172	24	83	2	39	0	110	3
Mycobacterium leprae	0	76	2	100	4	56	0	59	1	64	0	5	0	60	0
Mycobacterium tuberculosis CDC1551	1	124	2	118	4	123	2	133	18	102	0	8	0	92	0
Mycobacterium tuberculosis H37Rv	2	128	1	116	5	126	0	153	12	101	5	9	0	94	0
Mycoplasma genitalium G37	0	8	0	84	0	14	0	40	0	12	0	0	0	20	0
Mycoplasma pneumoniae M129	0	8	0	85	0	14	0	41	2	12	0	0	0	20	0
Mycoplasma pulmonis	0	5	2	84	3	19	2	52	8	8	0	0	0	17	1
Neisseria meningitidis serogroup A strain Z2491	0	42	0	122	3	59	0	125	6	127	1	27	0	63	0
Neisseria meningitidis serogroup B strain MC58	0	47	0	101	28	55	2	114	10	125	0	26	0	64	1
Nostoc sp. PCC 7120	0	65	1	134	0	98	3	139	10	186	2	26	0	153	3
Pasteurella multocida Pm70	0	41	0	132	0	77	2	92	7	141	0	8	0	86	0
Pseudomonas aeruginosa PAO1	2	155	2	157	0	191	2	123	7	211	0	124	3	162	2
Pyrobaculum aerophilum	0	43	0	124	0	59	0	72	0	34	0	6	0	65	0
Pyrococcus abyssi GE5	0	19	0	128	1	72	0	62	2	48	1	27	0	48	0
Pyrococcus horikoshii OT3	0	18	0	131	0	71	1	74	1	43	0	28	0	48	0
Ralstonia solanacearum	0	98	2	132	0	127	8	133	16	150	2	53	1	112	7
Rickettsia conorii Malish 7	1	30	3	107	1	23	0	61	4	75	3	4	0	53	2
Rickettsia prowazekii MadridE	0	27	0	106	0	17	0	54	0	73	0	2	0	50	0
Salmonella typhimurium LT2	1	81	1	143	1	183	7	130	18	228	8	81	3	136	5
Sinorhizobium meliloti 1021	1	74	2	130	2	85	2	104	7	110	3	46	0	104	2
Staphylococcus aureus subsp. Aureus N315	3	64	0	117	5	116	4	115	13	102	3	4	1	65	1
Streptococcus pneumoniae TIGR4	0	30	0	116	2	103	2	104	6	87	0	4	0	51	1
Streptococcus pyogenes	0	45	1	114	2	91	4	97	7	66	0	9	0	48	0
Sulfolobus solfataricus	0	69	4	127	1	82	1	116	26	54	8	14	0	65	2
Synechocystis sp PCC6803	3	51	0	121	3	61	5	98	17	128	11	27	2	118	2
Thermoplasma acidophilum	0	51	0	110	0	67	2	64	1	30	7	13	0	44	0
Thermoplasma volcanium	2	44	4	110	0	59	3	69	24	33	7	13	0	42	3
Thermotoga maritima MSB8	0	28	0	114	0	69	0	83	0	64	0	53	0	47	0
Treponema pallidum Nichols	1	17	0	98	1	26	0	58	1	63	1	42	0	40	1
Ureaplasma urealyticum serovar3	0	7	0	86	0	18	0	50	1	6	0	0	0	19	0
Vibrio cholerae chromosome I	0	60	2	133	0	110	8	105	12	128	8	81	8	92	2
Vibrio cholerae chromosome II	0	13	1	9	4	30	2	16	14	23	2	6	0	21	3
Xylella fastidiosa 9a5c	3	46	4	117	0	64	10	107	7	135	1	50	0	87	3
Yersinia pestis CO92	10	72	1	104	37	136	12	124	9	186	6	103	5	108	5

Table S7: Gene count in each of the COG categories (excluding the COG-None Categories)

DIMOB Islands (ORFs < 300bps excluded)

ORG	P_OUTSIDE	P_ISLAND	Q_OUTSIDE	Q_ISLAND	R_OUTSIDE	R_ISLAND	S_OUTSIDE	S_ISLAND	T_OUTSIDE	T_ISLAND	U_OUTSIDE	U_ISLAND	V_OUTSIDE	V_ISLAND
Aeropyrum pernix K1	46	0	10	0	201	0	108	0	11	0	7	0	18	0
Agrobacterium tumefaciens C58 Cereon Circular	122	2	29	0	246	6	242	6	75	1	25	0	25	0
Agrobacterium tumefaciens C58 Cereon Linear	73	0	26	0	133	8	116	1	24	1	5	1	12	0
Aquifex aeolicus VF5	53	2	18	0	153	1	93	0	38	0	18	0	9	0
Archaeoglobus fulgidus DSM4304	76	0	17	0	265	7	209	0	44	0	13	0	18	2
Bacillus halodurans C125	147	4	35	2	316	10	292	20	102	4	13	3	52	0
Bacillus subtilis 168	155	1	51	0	324	7	280	15	92	1	18	0	49	2
Borrelia burgdorferi B31	17	0	0	0	69	0	41	0	12	0	11	0	9	0
Brucella melitensis choromosome I	61	1	18	0	173	2	173	3	48	0	20	0	21	0
Brucella melitensis choromosome II	55	1	17	0	81	1	55	2	18	0	8	0	19	0
Buchnera sp. APS	15	0	1	0	26	0	14	0	4	0	11	0	4	0
Campylobacter jejuni NCTC11168	76	1	18	0	127	2	83	0	30	0	22	0	26	0
Caulobacter crescentus	105	4	66	0	271	7	302	4	78	0	44	1	38	2
Chlamydia trachomatis D	18	0	2	0	56	1	28	2	10	2	17	0	3	0
Chlamydophila pneumoniae CWL029	22	0	2	1	56	1	36	0	10	2	17	0	3	0
Clostridium acetobutylicum ATCC824	92	1	20	0	303	5	239	6	97	0	13	0	59	1
Corynebacterium glutamicum	136	3	30	1	217	6	158	6	51	3	14	0	31	1
Deinococcus radiodurans chrom 1	76	1	26	2	237	7	208	4	66	3	15	1	37	1
Deinococcus radiodurans chrom 2	18	1	9	0	23	1	13	0	12	0	0	0	11	0
Escherichia coli K12	167	16	42	2	320	18	271	12	91	2	35	0	46	2
Escherichia coli O157	198	7	41	1	364	33	343	31	97	6	35	6	52	3
Escherichia coli O157_EDL933	201	6	41	1	357	34	333	31	96	6	40	3	50	2
Fusobacterium nucleatum subsp. Nucleatum ATCC25586	78	0	10	0	171	0	118	0	25	0	23	0	32	0
Haemophilus influenzae Rd-KW20	71	0	12	0	129	2	109	1	29	0	23	0	16	0
Halobacterium sp. NRC1	72	3	17	1	190	4	134	8	30	1	8	0	20	0
Helicobacter pylori 26695	44	3	8	0	99	3	60	1	14	1	31	7	27	2
Helicobacter pylori J99	47	0	8	0	106	0	61	0	15	0	29	7	27	0
Lactococcus lactis	78	0	15	0	174	8	164	6	36	1	10	1	39	2
Listeria innocua Clip11262	107	0	26	0	251	4	243	8	79	0	16	0	56	2
Mesorhizobium loti	173	1	76	2	469	14	507	21	104	4	56	6	52	2
Methanobacterium thermoautotrophicum deltaH	65	0	8	0	223	0	195	1	27	3	10	0	16	0
Methanococcus jannaschii DSM2661	60	0	9	0	205	3	184	3	9	0	10	0	15	0
Methanopyrus kandleri AV19	29	0	9	0	200	0	180	0	13	0	10	0	5	0
Methanosarcina acetivorans str. C2A	152	4	21	4	368	17	309	15	53	0	20	1	56	3
Mycobacterium leprae	46	0	37	0	100	0	72	0	32	0	13	0	13	0
Mycobacterium tuberculosis CDC1551	109	1	82	1	290	4	210	6	80	1	15	1	37	0
Mycobacterium tuberculosis H37Rv	114	0	84	1	299	2	215	6	80	0	16	1	37	0
Mycoplasma genitalium G37	17	0	0	0	41	0	14	0	3	0	6	0	8	0
Mycoplasma pneumoniae M129	17	0	0	0	45	1	16	0	3	0	6	0	19	0
Mycoplasma pulmonis	16	0	0	0	50	2	24	1	3	0	8	0	29	0
Neisseria meningitidis serogroup A strain Z2491	75	4	12	3	145	0	147	1	21	2	22	0	14	2
Neisseria meningitidis serogroup B strain MC58	74	5	13	4	133	2	139	3	22	2	24	3	16	1
Nostoc sp. PCC 7120	203	3	71	2	360	6	315	5	133	1	30	0	68	6
Pasteurella multocida Pm70	112	4	16	1	177	1	152	3	43	0	34	0	26	1
Pseudomonas aeruginosa PAO1	261	0	90	0	451	6	432	3	135	2	65	0	64	0
Pyrobaculum aerophilum	57	0	12	0	239	0	140	0	21	0	7	0	22	0
Pyrococcus abyssi GE5	66	0	11	1	251	5	179	1	15	0	11	0	22	0
Pyrococcus horikoshii OT3	52	0	15	0	237	0	176	0	17	0	11	0	19	0
Ralstonia solanacearum	101	1	53	3	272	14	241	14	81	2	47	6	25	1
Rickettsia conorii Malish 7	19	1	8	0	75	1	50	4	16	1	29	0	20	0
Rickettsia prowazekii MadridE	19	0	10	0	59	0	41	0	12	0	29	0	9	0
Salmonella typhimurium LT2	166	2	48	0	333	32	290	21	90	0	38	0	45	1
Sinorhizobium meliloti 1021	105	2	28	1	241	9	273	7	30	0	33	0	22	3
Staphylococcus aureus subsp. Aureus N315	122	5	24	4	219	8	175	7	47	0	14	0	42	0
Streptococcus pneumoniae TIGR4	73	4	10	0	153	0	120	3	39	0	14	0	55	0
Streptococcus pyogenes	57	0	7	0	132	4	117	3	39	0	12	0	37	2
Sulfolobus solfataricus	57	1	30	1	266	27	145	16	13	1	10	0	21	0
Synechocystis sp PCC6803	142	5	35	1	249	12	183	4	98	10	23	0	38	1
Thermoplasma acidophilum	33	0	13	3	148	6	98	3	9	0	10	0	13	1
Thermoplasma volcanium	31	1	11	2	138	8	94	3	9	1	9	0	11	5
Thermotoga maritima MSB8	64	0	13	0	188	0	118	0	46	0	11	0	31	0
Treponema pallidum Nichols	23	0	2	0	78	1	45	2	21	0	11	0	6	1
Ureaplasma urealyticum serovar3	24	0	0	0	45	0	27	0	3	0	6	0	15	0
Vibrio cholerae chromosome I	97	0	31	3	194	6	166	8	91	2	32	0	34	3
Vibrio cholerae chromosome II	50	1	7	0	77	7	63	7	22	0	4	0	7	3
Xylella fastidiosa 9a5c	60	1	20	0	174	11	145	8	44	0	43	0	30	0
Yersinia pestis CO92	173	11	39	5	246	9	293	24	76	3	39	15	38	4

Table S7: Gene count in each of the COG categories (excluding the COG-None Categories)

INFORMATION STORAGE AND PROCESSING
[J] Translation, ribosomal structure and biogenesis
[A] RNA processing and modification
[K] Transcription
[L] Replication, recombination and repair
[B] Chromatin structure and dynamics
CELLULAR PROCESSES AND SIGNALING
[D] Cell cycle control, cell division, chromosome partitioning
[V] Defense mechanisms
[T] Signal transduction mechanisms
[M] Cell wall/membrane/envelope biogenesis
[N] Cell motility
[U] Intracellular trafficking, secretion, and vesicular transport
[O] Posttranslational modification, protein turnover, chaperones
METABOLISM
[C] Energy production and conversion
[G] Carbohydrate transport and metabolism
[E] Amino acid transport and metabolism
[F] Nucleotide transport and metabolism
[H] Coenzyme transport and metabolism
[I] Lipid transport and metabolism
[P] Inorganic ion transport and metabolism
[Q] Secondary metabolites biosynthesis, transport and catabolism
POORLY CHARACTERIZED
[R] General function prediction only
[S] Function unknown
