# Supplementary material for: Evidence of a Large Novel Gene Pool Associated with Prokaryotic Genomic Islands
Source: PLoS Genet. 2005 Nov 18;1(5):e62. doi: 10.1371/journal.pgen.0010062 (PMC1285063; doi:10.1371/journal.pgen.0010062)
Supplement: Supplementary file 4 [file pgen.0010062.st008.pdf]

Table S8: Summary of gene counts in islands and outside of islands (ORFS < 300bps excluded)
Headings: Novel_Island= number of novel genes in islands; Total_Island= number of genes in islands; Novel_Outside= number of nove lgenes outside of islands; Total_Outside= number of genes outside of islands

DINUC Islands, COG Novel Gnes

ORG	Novel_Island	Total_Island	Novel_Outside	Total_Outside
Aeropyrum pernix K1	159	310	419	1443
Agrobacterium tumefaciens C58 Cereon Circular	64	296	331	2221
Agrobacterium tumefaciens C58 Cereon Linear	73	156	649	1567
Aquifex aeolicus VF5	31	219	167	1270
Archaeoglobus fulgidus DSM4304	90	273	331	1829
Bacillus halodurans C125	114	386	719	3174
Bacillus subtilis 168	205	433	699	3197
Borrelia burgdorferi B31	30	140	143	633
Brucella melitensis choromosome I	56	186	176	1638
Brucella melitensis choromosome II	32	131	195	896
Buchnera sp. APS	6	74	20	449
Campylobacter jejuni NCTC11168	38	162	208	1323
Caulobacter crescentus	143	505	815	2952
Chlamydia trachomatis D	42	134	174	699
Chlamydophila pneumoniae CWL029	58	131	270	838
Clostridium acetobutylicum ATCC824	176	450	718	2818
Corynebacterium glutamicum	195	366	733	2380
Deinococcus radiodurans chrom 1	113	303	548	2168
Deinococcus radiodurans chrom 2	28	49	94	309
Escherichia coli K12	136	451	593	3448
Escherichia coli O157	397	755	816	3868
Escherichia coli O157_EDL933	369	703	853	3918
Fusobacterium nucleatum subsp. Nucleatum ATCC25586	71	285	339	1562
Haemophilus influenzae Rd-KW20	23	161	173	1367
Halobacterium sp. NRC1	79	264	307	1524
Helicobacter pylori 26695	57	116	285	1286
Helicobacter pylori J99	54	104	271	1267
Lactococcus lactis	113	282	357	1747
Listeria innocua Clip11262	182	392	370	2296

Mesorhizobium loti	367	800	1851	5265
Methanobacterium thermoautotrophicum deltaH	66	235	221	1413
Methanococcus jannaschii DSM2661	74	254	200	1301
Methanopyrus kandleri AV19	96	271	296	1281
Methanosarcina acetivorans str. C2A	305	622	1218	3361
Mycobacterium leprae	34	151	302	1310
Mycobacterium tuberculosis CDC1551	223	449	1201	3203
Mycobacterium tuberculosis H37Rv	200	426	1209	3269
Mycoplasma genitalium G37	16	84	81	379
Mycoplasma pneumoniae M129	57	102	199	561
Mycoplasma pulmonis	34	102	185	604
Neisseria meningitidis serogroup A strain Z2491	76	183	205	1552
Neisseria meningitidis serogroup B strain MC58	96	199	207	1549
Nostoc sp. PCC 7120	300	727	1600	3933
Pasteurella multocida Pm70	38	189	123	1717
Pseudomonas aeruginosa PAO1	177	639	1325	4618
Pyrobaculum aerophilum	160	279	601	1866
Pyrococcus abyssi GE5	48	240	236	1453
Pyrococcus horikoshii OT3	71	197	310	1525
Ralstonia solanacearum	145	452	551	2671
Rickettsia conorii Malish 7	35	130	187	868
Rickettsia prowazekii MadridE	19	106	74	666
Salmonella typhimurium LT2	206	527	625	3520
Sinorhizobium meliloti 1021	181	381	818	2689
Staphylococcus aureus subsp. Aureus N315	100	243	326	2054
Streptococcus pneumoniae TIGR4	32	125	226	1584
Streptococcus pyogenes	66	182	174	1329
Sulfolobus solfataricus	247	441	757	2316
Synechocystis sp PCC6803	133	295	755	2617
Thermoplasma acidophilum	58	216	191	1159
Thermoplasma volcanium	59	225	189	1144
Thermotoga maritima MSB8	49	229	198	1467
Treponema pallidum Nichols	47	140	183	780
Ureaplasma urealyticum serovar3	38	68	133	494
Vibrio cholerae chromosome I	59	267	282	2104
Vibrio cholerae chromosome II	81	148	226	723

Xylella fastidiosa 9a5c	113	268	347	1773
Yersinia pestis CO92	116	401	624	3114

DIMOB islands, COG Novel Genes

ORG	Novel_Island	Total_Island	Novel_Outside	Total_Outside
Agrobacterium tumefaciens C58 Cereon Circular	19	50	376	2467
Agrobacterium tumefaciens C58 Cereon Linear	34	63	688	1660
Aquifex aeolicus VF5	1	21	197	1468
Archaeoglobus fulgidus DSM4304	10	28	411	2074
Bacillus halodurans C125	57	185	776	3375
Bacillus subtilis 168	129	193	775	3437
Brucella melitensis choromosome I	45	101	187	1723
Brucella melitensis choromosome II	10	25	217	1002
Campylobacter jejuni NCTC11168	3	9	243	1476
Caulobacter crescentus	33	80	925	3377
Chlamydia trachomatis D	5	12	211	821
Chlamydophila pneumoniae CWL029	9	19	319	950
Clostridium acetobutylicum ATCC824	44	71	850	3197
Corynebacterium glutamicum	72	114	856	2632
Deinococcus radiodurans chrom 1	31	67	630	2404
Deinococcus radiodurans chrom 2	14	17	108	341
Escherichia coli K12	106	253	623	3646
Escherichia coli O157	271	432	942	4191
Escherichia coli O157_EDL933	271	443	951	4178
Fusobacterium nucleatum subsp. Nucleatum ATCC25586	5	9	405	1838
Haemophilus influenzae Rd-KW20	5	11	191	1517
Halobacterium sp. NRC1	44	115	342	1673
Helicobacter pylori 26695	23	57	319	1345
Helicobacter pylori J99	19	35	306	1336
Lactococcus lactis	52	121	418	1908
Listeria innocua Clip11262	63	97	489	2591
Mesorhizobium loti	145	286	2073	5779
Methanobacterium thermoautotrophicum deltaH	0	8	287	1640
Methanococcus jannaschii DSM2661	6	23	268	1532
Methanosarcina acetivorans str. C2A	105	220	1418	3763
Mycobacterium leprae	2	10	334	1451

Mycobacterium tuberculosis CDC1551	51	99	1373	3553
Mycobacterium tuberculosis H37Rv	40	77	1369	3618
Mycoplasma pneumoniae M129	4	8	252	655
Mycoplasma pulmonis	14	33	205	673
Neisseria meningitidis serogroup A strain Z2491	18	48	263	1687
Neisseria meningitidis serogroup B strain MC58	33	97	270	1651
Nostoc sp. PCC 7120	47	100	1853	4560
Pasteurella multocida Pm70	8	30	153	1876
Pseudomonas aeruginosa PAO1	20	52	1482	5205
Pyrococcus abyssi GE5	6	19	278	1674
Pyrococcus horikoshii OT3	5	8	376	1714
Ralstonia solanacearum	66	152	630	2971
Rickettsia conorii Malish 7	12	36	210	962
Salmonella typhimurium LT2	114	228	717	3819
Sinorhizobium meliloti 1021	83	130	916	2940
Staphylococcus aureus subsp. Aureus N315	25	88	401	2209
Streptococcus pneumoniae TIGR4	10	32	248	1677
Streptococcus pyogenes	24	50	216	1461
Sulfolobus solfataricus	213	321	791	2436
Synechocystis sp PCC6803	97	190	791	2722
Thermoplasma acidophilum	12	35	237	1340
Thermoplasma volcanium	49	125	199	1244
Treponema pallidum Nichols	3	15	227	905
Ureaplasma urealyticum serovar3	23	24	148	538
Vibrio cholerae chromosome I	49	120	292	2251
Vibrio cholerae chromosome II	49	99	258	772
Xylella fastidiosa 9a5c	48	112	412	1929
Yersinia pestis CO92	74	245	666	3270

DINUC Islands, SUPERFAMILY Novel Genes

ORG	Novel Island	Total_Island	Novel_Outside	Total_Outside
Aeropyrum pernix K1	166	310	578	1443
Agrobacterium tumefaciens C58 Cereon Circular	112	296	684	2221
Agrobacterium tumefaciens C58 Cereon Linear	57	156	427	1567
Aquifex aeolicus VF5	85	219	341	1270
Archaeoglobus fulgidus DSM4304	124	273	541	1829

Bacillus halodurans C125	157	386	1061	3174
Bacillus subtilis 168	206	433	1044	3197
Borrelia burgdorferi B31	52	140	221	633
Brucella melitensis choromosome I	57	186	488	1638
Brucella melitensis choromosome II	61	131	236	896
Buchnera sp. APS	15	74	77	449
Campylobacter jejuni NCTC11168	52	162	436	1323
Caulobacter crescentus	192	505	915	2952
Chlamydia trachomatis D	58	134	254	699
Chlamydophila pneumoniae CWL029	55	131	347	838
Clostridium acetobutylicum ATCC824	177	450	972	2818
Corynebacterium glutamicum	183	366	802	2380
Deinococcus radiodurans chrom 1	117	303	755	2168
Deinococcus radiodurans chrom 2	25	49	92	309
Escherichia coli K12	183	451	1314	3448
Escherichia coli O157	371	755	1298	3868
Escherichia coli O157_EDL933	341	703	1328	3918
Fusobacterium nucleatum subsp. Nucleatum ATCC25586	106	285	524	1562
Haemophilus influenzae Rd-KW20	52	161	396	1367
Halobacterium sp. NRC1	107	264	488	1524
Helicobacter pylori 26695	72	116	480	1286
Helicobacter pylori J99	66	104	464	1267
Lactococcus lactis	117	282	535	1747
Listeria innocua Clip11262	192	392	696	2296
Mesorhizobium loti	320	800	1641	5265
Methanobacterium thermoautotrophicum deltaH	105	235	416	1413
Methanococcus jannaschii DSM2661	99	254	413	1301
Methanopyrus kandleri AV19	129	271	412	1281
Methanosarcina acetivorans str. C2A	247	622	1196	3361
Mycobacterium leprae	42	151	399	1310
Mycobacterium tuberculosis CDC1551	216	449	1118	3203
Mycobacterium tuberculosis H37Rv	186	426	1112	3269
Mycoplasma genitalium G37	18	84	122	379
Mycoplasma pneumoniae M129	52	102	226	561
Mycoplasma pulmonis	41	102	223	604
Neisseria meningitidis serogroup A strain Z2491	86	183	498	1552

Neisseria meningitidis serogroup B strain MC58	104	199	486	1549
Nostoc sp. PCC 7120	288	727	1386	3933
Pasteurella multocida Pm70	68	189	520	1717
Pseudomonas aeruginosa PAO1	239	639	1378	4618
Pyrobaculum aerophilum	147	279	718	1866
Pyrococcus abyssi GE5	107	240	468	1453
Pyrococcus horikoshii OT3	100	197	551	1525
Ralstonia solanacearum	171	452	832	2671
Rickettsia conorii Malish 7	56	130	343	868
Rickettsia prowazekii MadridE	37	106	200	666
Salmonella typhimurium LT2	222	527	1151	3520
Sinorhizobium meliloti 1021	132	381	780	2689
Staphylococcus aureus subsp. Aureus N315	100	243	672	2054
Streptococcus pneumoniae TIGR4	40	125	447	1584
Streptococcus pyogenes	81	182	382	1329
Sulfolobus solfataricus	228	441	837	2316
Synechocystis sp PCC6803	116	295	866	2617
Thermoplasma acidophilum	79	216	360	1159
Thermoplasma volcanium	89	225	334	1144
Thermotoga maritima MSB8	69	229	419	1467
Treponema pallidum Nichols	62	140	285	780
Ureaplasma urealyticum serovar3	36	68	175	494
Vibrio cholerae chromosome I	76	267	654	2104
Vibrio cholerae chromosome II	84	148	248	723
Xylella fastidiosa 9a5c	119	268	619	1773
Yersinia pestis CO92	167	401	1090	3114

DIMOB Islands, SUPERFAMILY Novel Genes

Organism Name	Novel_Island	Total_Island	Novel_Outside	Total_Outside
Agrobacterium tumefaciens C58 Cereon Circular	27	50	769	2467
Agrobacterium tumefaciens C58 Cereon Linear	18	63	466	1660
Aquifex aeolicus VF5	4	21	422	1468
Archaeoglobus fulgidus DSM4304	16	28	649	2074
Bacillus halodurans C125	67	185	1151	3375
Bacillus subtilis 168	120	193	1130	3437
Brucella melitensis choromosome I	41	101	504	1723

Brucella melitensis choromosome II	13	25	284	1002
Campylobacter jejuni NCTC11168	3	9	485	1476
Caulobacter crescentus	35	80	1072	3377
Chlamydia trachomatis D	7	12	305	821
Chlamydophila pneumoniae CWL029	9	19	393	950
Clostridium acetobutylicum ATCC824	39	71	1110	3197
Corynebacterium glutamicum	63	114	922	2632
Deinococcus radiodurans chrom 1	29	67	843	2404
Deinococcus radiodurans chrom 2	11	17	106	341
Escherichia coli K12	124	253	1373	3646
Escherichia coli O157	238	432	1431	4191
Escherichia coli O157_EDL933	237	443	1432	4178
Fusobacterium nucleatum subsp. Nucleatum ATCC25586	4	9	626	1838
Haemophilus influenzae Rd-KW20	6	11	442	1517
Halobacterium sp. NRC1	54	115	541	1673
Helicobacter pylori 26695	31	57	521	1345
Helicobacter pylori J99	24	35	506	1336
Lactococcus lactis	46	121	606	1908
Listeria innocua Clip11262	49	97	839	2591
Mesorhizobium loti	122	286	1839	5779
Methanobacterium thermoautotrophicum deltaH	0	8	521	1640
Methanococcus jannaschii DSM2661	7	23	505	1532
Methanosarcina acetivorans str. C2A	83	220	1360	3763
Mycobacterium leprae	2	10	439	1451
Mycobacterium tuberculosis CDC1551	60	99	1274	3553
Mycobacterium tuberculosis H37Rv	33	77	1265	3618
Mycoplasma pneumoniae M129	5	8	273	655
Mycoplasma pulmonis	15	33	249	673
Neisseria meningitidis serogroup A strain Z2491	26	48	558	1687
Neisseria meningitidis serogroup B strain MC58	47	97	543	1651
Nostoc sp. PCC 7120	41	100	1633	4560
Pasteurella multocida Pm70	15	30	573	1876
Pseudomonas aeruginosa PAO1	24	52	1593	5205
Pyrococcus abyssi GE5	7	19	568	1674
Pyrococcus horikoshii OT3	2	8	649	1714
Ralstonia solanacearum	75	152	928	2971

Rickettsia conorii Malish 7	20	36	379	962
Salmonella typhimurium LT2	123	228	1250	3819
Sinorhizobium meliloti 1021	49	130	863	2940
Staphylococcus aureus subsp. Aureus N315	29	88	743	2209
Streptococcus pneumoniae TIGR4	13	32	474	1677
Streptococcus pyogenes	27	50	436	1461
Sulfolobus solfataricus	179	321	886	2436
Synechocystis sp PCC6803	76	190	906	2722
Thermoplasma acidophilum	15	35	424	1340
Thermoplasma volcanium	56	125	367	1244
Treponema pallidum Nichols	5	15	342	905
Ureaplasma urealyticum serovar3	19	24	192	538
Vibrio cholerae chromosome I	50	120	680	2251
Vibrio cholerae chromosome II	55	99	277	772
Xylella fastidiosa 9a5c	51	112	687	1929
Yersinia pestis CO92	95	245	1162	3270
