# Supplementary material for: Evidence of a Large Novel Gene Pool Associated with Prokaryotic Genomic Islands
Source: PLoS Genet. 2005 Nov 18;1(5):e62. doi: 10.1371/journal.pgen.0010062 (PMC1285063; doi:10.1371/journal.pgen.0010062)
Supplement: Supplementary file 5 [file pgen.0010062.sd001.doc]

Protocol S1:

Because of the consistently higher proportions of None-category genes in islands, the percentages of genes in other categories may be correspondingly depressed. Therefore, we removed all the None-category genes from the total gene set when calculating the distribution of genes in the other COG categories discussed here below. As expected in the DINUC dataset, genes in the translation, ribosomal structure and biogenesis (category J) were over-represented in dinucleotide biased regions (p-value = 6.3E-11) due to that fact that ribosomal proteins are noted for exhibiting unusual gene composition. In the DIMOB dataset where we took the presence of mobility genes into account, the difference between the number of genes in islands versus outside of islands for this category becomes insignificant (α = 0.01). Also, as expected, genes involved in replication, recombination and repair (Category L) are present in a significantly higher proportion inside islands vs. outside islands. This category includes all mobility genes (e.g. transposases, integrases, recombinases). The existence of this correlation in the set based on dinucleotide bias alone supports the association of dinucleotide bias with horizontally acquired regions.

Conversely, genes involved in macromolecule biosynthesis (transport and metabolism genes for lipid, amino acid, nucleotide, carbohydrate, and co-enzymes) are present in significantly lower proportions in the predicted islands versus outside of islands. We also found that there is no difference in the proportion of transcriptional genes in islands vs. outside of islands. This result may appear to contradict observations made by Jain *et al.* (46); however, the differences are likely due more to differences in the type of HGT being detected. Their data set from six organisms consisted of a rather small set of homologous genes which were more likely to be subject to orthologous displacement than to de-novo acquisition. Since sequence compositional approaches are less able to detect orthologous displacement from organisms with similar compositions, our result suggests that if these genes have indeed undergone HGT, the mode of transfer is likely to be homologous recombination between closely related species or ancient HGT that has been subject to amelioration.
